# PKA Controls Calcium Influx into Motor Neurons during a Rhythmic Behavior

**DOI:** 10.1371/journal.pgen.1003831

**Published:** 2013-09-26

**Authors:** Han Wang, Derek Sieburth

**Affiliations:** 1 Graduate Program in Genetic, Molecular and Cellular Biology, Keck School of Medicine, University of Southern California, Los Angeles, California, United States of America; 2 Zilkha Neurogenetic Institute, Department of Cell and Neurobiology, Keck School of Medicine, University of Southern California, Los Angeles, California, United States of America; Stanford University School of Medicine, United States of America

## Abstract

Cyclic adenosine monophosphate (cAMP) has been implicated in the execution of diverse rhythmic behaviors, but how cAMP functions in neurons to generate behavioral outputs remains unclear. During the defecation motor program in *C. elegans*, a peptide released from the pacemaker (the intestine) rhythmically excites the GABAergic neurons that control enteric muscle contractions by activating a G protein-coupled receptor (GPCR) signaling pathway that is dependent on cAMP. Here, we show that the *C. elegans* PKA catalytic subunit, KIN-1, is the sole cAMP target in this pathway and that PKA is essential for enteric muscle contractions. Genetic analysis using cell-specific expression of dominant negative or constitutively active PKA transgenes reveals that knockdown of PKA activity in the GABAergic neurons blocks enteric muscle contractions, whereas constitutive PKA activation restores enteric muscle contractions to mutants defective in the peptidergic signaling pathway. Using real-time, in vivo calcium imaging, we find that PKA activity in the GABAergic neurons is essential for the generation of synaptic calcium transients that drive GABA release. In addition, constitutively active PKA increases the duration of calcium transients and causes ectopic calcium transients that can trigger out-of-phase enteric muscle contractions. Finally, we show that the voltage-gated calcium channels UNC-2 and EGL-19, but not CCA-1 function downstream of PKA to promote enteric muscle contractions and rhythmic calcium influx in the GABAergic neurons. Thus, our results suggest that PKA activates neurons during a rhythmic behavior by promoting presynaptic calcium influx through specific voltage-gated calcium channels.

## Author Summary

Breathing, walking and sleeping, are examples of rhythmic behaviors that occur at regular time intervals. The time intervals are determined by pacemakers, which generate the rhythms, and the behaviors are carried out by different tissues such as neurons and muscles. How do timing signals from pacemakers get delivered to target tissues to ensure proper execution of these behaviors? To begin to address this question, we study a simple rhythmic behavior in the nematode *C. elegans* called the defecation motor program. In this behavior, enteric muscles contract every 50 seconds, allowing digested food to be expelled from the gut. The pacemaker is the gut itself, and here we identify a specific protein, PKA, that responds to the signal from the pacemaker by activating certain neurons that trigger enteric muscle contraction. We further demonstrate that PKA activates these neurons by controlling the entry of calcium into these neurons. We also identify two calcium channels that allow calcium to enter the neurons when PKA is activated by the signal from the pacemaker. Our results raise the possibility that PKA-mediated calcium entry might be a mechanism used in other organisms to regulate rhythmic behaviors.

## Introduction

Cyclic adenosine monophosphate (cAMP) is a potent second messenger that plays an important role in cellular responses to extracellular signals to regulate a wide array of biological processes. In the nervous system, cAMP has been implicated in controlling axon guidance, axonal regeneration, sensory function, learning and memory [1]–[4]. cAMP signaling is also critical for the execution of rhythmic physiological processes such as heart beating and circadian rhythm in a variety of organisms [5]–[8]. However, the mechanism by which cAMP controls rhythmic outputs remains unclear.

**Figure 1:**
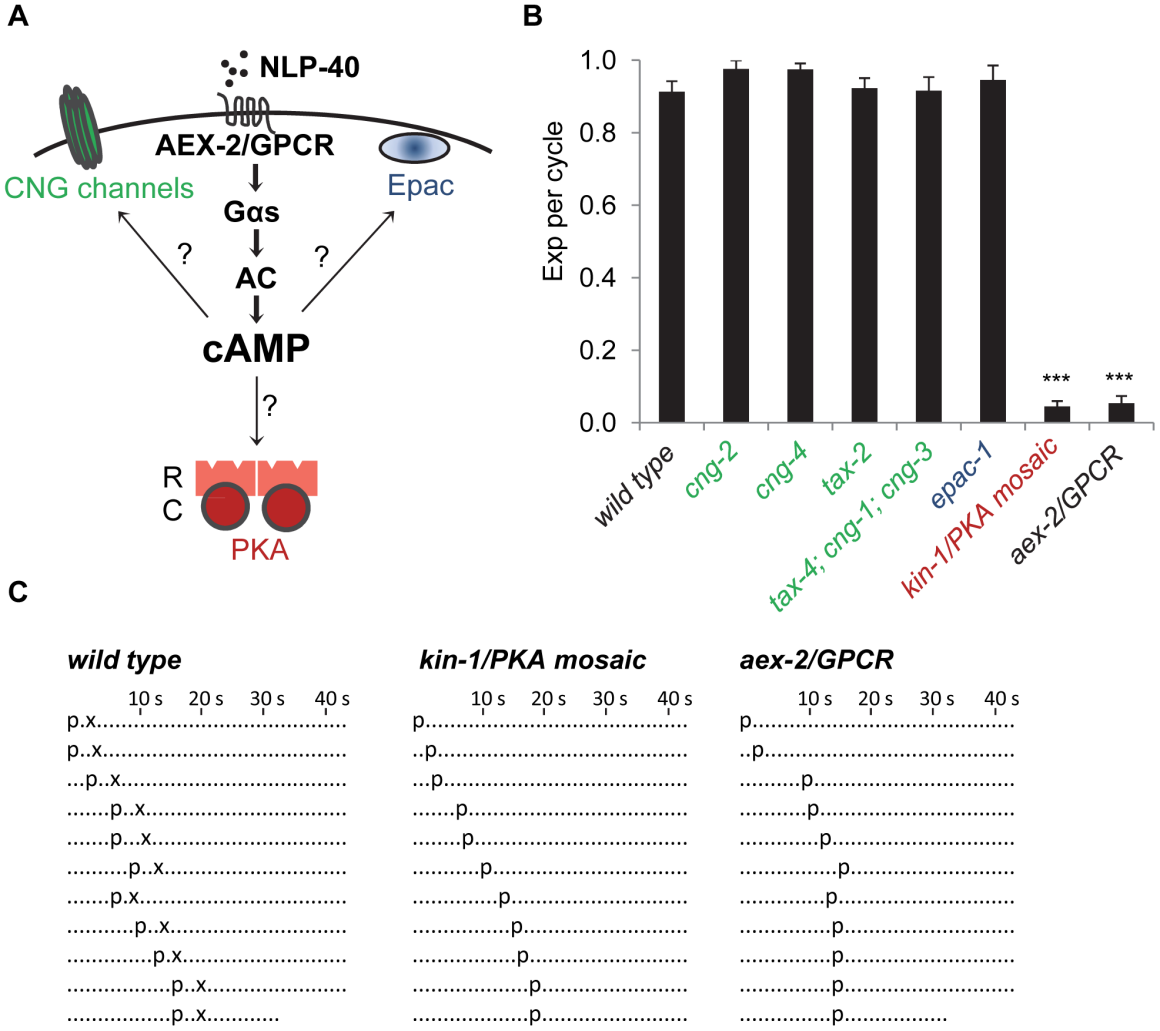
PKA activity is essential for the Exp step. (A) Diagram of the NLP-40-AEX-2/GPCR peptidergic signaling pathway acting in the GABAergic neurons that controls rhythmic release of GABA during the enteric muscle contraction (Exp) step of the defecation motor program. The potential cAMP targets in this peptidergic pathway are indicated. The PKA holoenzyme is a tetramer with two regulatory subunits (R) and two catalytic subunits (C). (B) Quantification of the Exp step in young adult worms with the indicated genotypes. “Exp per cycle” is defined as the average of the ratio of Exp to pBoc. (C) Representative ethograms of ten consecutive defecation cycles of young adult worms with the indicated genotypes. The *kin-1* mosaic represents constipated worms segregated from the strain DG3393: *kin-1(ok338)* I; *tnEx109*. Each dot represents 1 s. “p” stands for the pBoc step and “x” indicates the Exp step. aBoc is omitted. Means and standard errors are shown. Asterisks (***) indicate significant difference from wild type: p<0.005 in Student's t-test.

cAMP is synthesized by adenylyl cyclases (ACs), which are activated by G protein-coupled receptors (GPCRs) that are coupled to the heterotrimeric G protein α subunit, Gαs [9]. Work in a variety of cell types has shown that cAMP has three major molecular targets: cyclic nucleotide-gated (CNG) channels, exchange proteins directly activated by cAMP (Epac) and cAMP-dependent protein kinase (PKA) (Figure 1A and [9]). CNG channels are non-selective cation channels that are critical for the excitability of certain sensory neurons [10]. Epac proteins are guanine exchange factors for the small G protein Rap, and have been shown to regulate cardiac function and insulin secretion [11]. PKA is a conserved serine/threonine kinase that has been implicated in a wide array of biological processes, including cell growth, neural function, cell differentiation and metabolism [12]. In neurons and neurosecretory cells, PKA regulates the release of neurotransmitter and neuropeptides [13]. PKA activity has also been implicated in the execution of rhythmic behaviors, such as sleep and circadian locomotor activity in the fly [14], [15].

PKA phosphorylates many substrates in excitable cells. For example, in cardiac muscles, PKA phosphorylates the ryanodine receptor and the L-type calcium channel to regulate heart beating [8], [16]. In neurons, several synaptic proteins, such as RIM-1α, synapsin and tomosyn are reported as PKA substrates that regulate neurotransmitter release [13], [17]. In addition, it has been shown that PKA can phosphorylate calcium channels in hippocampal neurons, which may account for PKA-dependent modulation of neurotransmitter release and gene expression [18]. However, it is unclear how PKA impacts the physiology of neurons to regulate rhythmic behavioral outputs.

The *C. elegans* defecation motor program is a simple rhythmic behavior that occurs about every 50 seconds [19]. Each cycle contains three sequential muscle contractions: the posterior body wall muscle contraction (pBoc), anterior body wall muscle contraction (aBoc), and enteric muscle contraction (Exp, which leads to the expulsion of the gut contents). The period of the defecation cycle is controlled by a pacemaker in the intestine, and the Exp step is initiated by the release of a neuropeptide-like protein NLP-40 from the intestine. NLP-40 instructs the excitation of a pair of GABAergic neurons (AVL and DVB), which in turn release the neurotransmitter GABA to trigger the Exp step [20]–[23]. NLP-40 activates the GPCR AEX-2 on the GABAergic neurons, which is coupled to the heterotrimeric G protein α subunit GSA-1/Gαs, leading to the activation of adenylyl cyclase and the production of cAMP [5], [23]. However, the molecular targets of cAMP in the GABAergic neurons are not known and how cAMP signaling impacts GABA release to mediate the Exp step is unclear.

In this study, we find that KIN-1, the *C. elegans* homolog of the PKA catalytic subunit, is essential for the rhythmic contraction of enteric muscles. By genetically manipulating the activity of PKA specifically in the GABAergic neurons, we establish that PKA is the downstream target of cAMP in the peptidergic signaling pathway that controls enteric muscle contraction. Furthermore, using in vivo calcium imaging, we find that PKA activates the DVB neuron by promoting calcium influx at presynaptic terminals, and that the voltage-gated calcium channels, UNC-2 and EGL-19, partially mediate PKA-dependent calcium influx. Thus, our results suggest that PKA signaling can control rhythmic behaviors by regulating presynaptic calcium entry.

## Results

### PKA regulates the Exp step

To identify cAMP effectors that mediate enteric muscle contraction, we first examined *C. elegans* mutant worms of putative cAMP targets for defects in the Exp step. The *C. elegans* genome encodes six CNG channels: *cng-1*, *cng-2*, *cng-3*, *cng-4/che-6*, *tax-2* and *tax-4* [24], some of which have well characterized roles in sensory transduction [10], whereas the functions of the other channels are largely unknown. Putative null or loss-of-function mutations in any of these CNGs caused no obvious defects in the defecation motor program period or in the execution of pBoc or Exp (Figure 1B and data not shown). The *C. elegans* genome encodes a single Epac homolog, *epac-1*. Putative null *epac-1* mutants had no defects in the defecation motor program, including in the Exp step (Figure 1B and data not shown). Thus, CNG channels and Epac are unlikely to be the cAMP targets that control the execution of the Exp step.

The PKA catalytic subunit is encoded by a single gene, *kin-1*, in *C. elegans*. *kin-1(ok338)* loss-of-function mutants, which contain a 763 bp deletion in *kin-1* that removes part of the catalytic domain, die during embryogenesis. The lethality of *kin-1(ok338)* animals can be partially rescued by mosaic expression of wild type *kin-1* transgenes [25]. We found that a fraction of mosaic *kin-1(ok338)* animals that survive to adulthood had distended intestinal lumens indicative of constipation. These animals lacked the Exp step in nearly all defecation cycles but both execution of pBoc and cycle length were normal (Figure 1B and 1C and data not shown). The Exp defects seen in constipated *kin-1* mosaic worms were almost identical to those of animals lacking *nlp-40 or aex-2/GPCR*, which are components of the peptidergic signaling pathway that activates the GABAergic neurons (Figure 1B and 1C and [23]). Taken together, we conclude that PKA activity is absolutely required for the generation of rhythmic enteric muscle contractions and that PKA is likely to be a major, if not the only, downstream target of cAMP during the Exp step of the defecation motor program.

### PKA functions in GABAergic neurons to promote the Exp step

**Figure 2:**
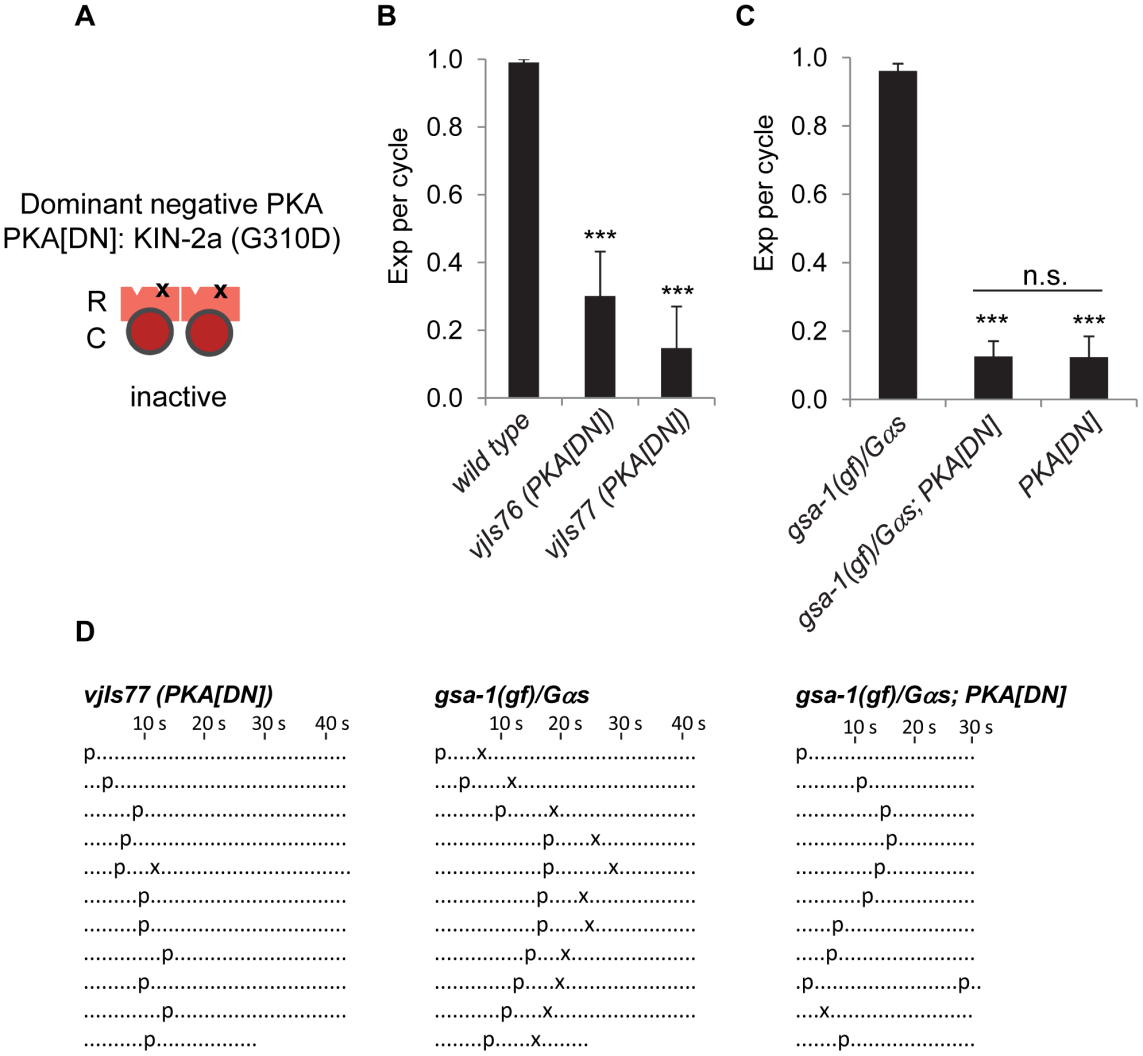
PKA functions in GABAergic neurons to regulate the Exp step. (A) Diagram showing the construction of dominant negative PKA (PKA[DN]). “R” and “C” indicate the PKA regulatory and catalytic subunit, respectively. “x” represents the substitution (G310D) in the site B of the regulatory subunit KIN-2a, which presumably blocks cAMP binding and prevents its dissociation with PKA catalytic subunit. (B) and (C) Quantification of the Exp step of young adults with the indicated genotypes. PKA[DN] denotes PKA dominant negative transgenic worms (*vjIs76* and *vjIs77*) in which the mutated regulatory subunit *kin-2a*(G310D) was expressed specifically in GABAergic neurons using *unc-47* full length promoter. *gsa-1(gf)* is a gain-of-function allele (*ce81)* of *gsa-1*/Gαs. (D) Representative ethograms of ten consecutive defecation cycles of young adult worms with the indicated genotypes. *vjIs77* is used for PKA[DN] in (D). Each dot represents 1 s. “p” stands for the pBoc step and “x” indicates the Exp step. aBoc is omitted. Means and standard errors are shown. Asterisks (***) indicate significant difference from wild type in (B) and *gas-1(gf)/Gas* in (C): p<0.005 in Student's t-test. “n.s.” indicates no significant difference between indicated groups.

To determine whether PKA functions in GABAergic neurons to control the Exp step, we generated worms expressing dominant negative PKA transgenes specifically in these neurons. The PKA holoenzyme is a tetramer composed of two regulatory (R) subunits and two catalytic (C) subunits (Figure 1A). When cAMP levels are low, R subunits bind to and inhibit the activity of C subunits; when cAMP levels increase, two cAMP molecules bind to each of the R subunits at two distinct sites (site A and site B), leading to the dissociation of the PKA holoenzyme and activation of the C subunits [12]. It has been shown that a single amino acid substitution (G324D) in site B of the mouse regulatory subunit (RIα) abolishes cAMP binding and prevents the dissociation of the PKA holoenzyme in vitro [26]. Expression of this mutant R subunit in mice generates a dominant negative effect on PKA activity in vivo [27]. *kin-2* encodes the sole R subunit in *C. elegans*, and KIN-2 shares 74% overall sequence similarity with mouse RIα, and 97% similarity in the cAMP binding sites (Figure S1). We mutated the corresponding Gly residue to Asp (G310D) in the B site of the KIN-2a isoform (referred to as PKA[DN], Figure 2A and S1) and expressed this construct specifically in GABAergic motor neurons (using the full length *unc-47* promoter) [5], [23]. Two independently generated PKA[DN] transgenic lines (*vjIs76* and *vjIs77*) displayed distended intestinal lumens, and dramatically reduced cycles in which Exp occurred (Figure 2B and 2D and data not shown). This phenotype was similar to, but not as severe as that of the mosaic *kin-1* mutants, likely due to variable expression of the *unc-47* promoter in DVB neurons (data not shown). Thus, PKA activity is required in GABAergic neurons to promote the Exp step.

### PKA acts downstream of Gαs in the NLP-40-AEX-2/GPCR peptidergic signaling pathway

To determine whether PKA functions in the peptidergic signaling pathway activated in the GABAergic neurons that control the Exp step, we examined whether PKA[DN] transgenes could block Exp in animals in which this pathway is constitutively active. *gsa-1/*Gαs gain-of-function (*gsa-1(gf)*) mutations restore Exp to *aex-2*/GPCR mutants, consistent with a role for *gsa-1/*Gαs downstream of *aex-2*/GPCR [5]. However, *gsa-1(gf)* mutations failed to restore Exp to animals expressing PKA[DN] (Figure 2C and 2D). These data show that the effects of the AEX-2/GPCR peptidergic signaling pathway on Exp are dependent on PKA in the GABAergic neurons. For reasons that are unclear, the *gsa-1(ce81); PKA[DN]* double mutants had a slightly shorter cycle length compared with either single mutant (Figure 2D and data not shown).

**Figure 3:**
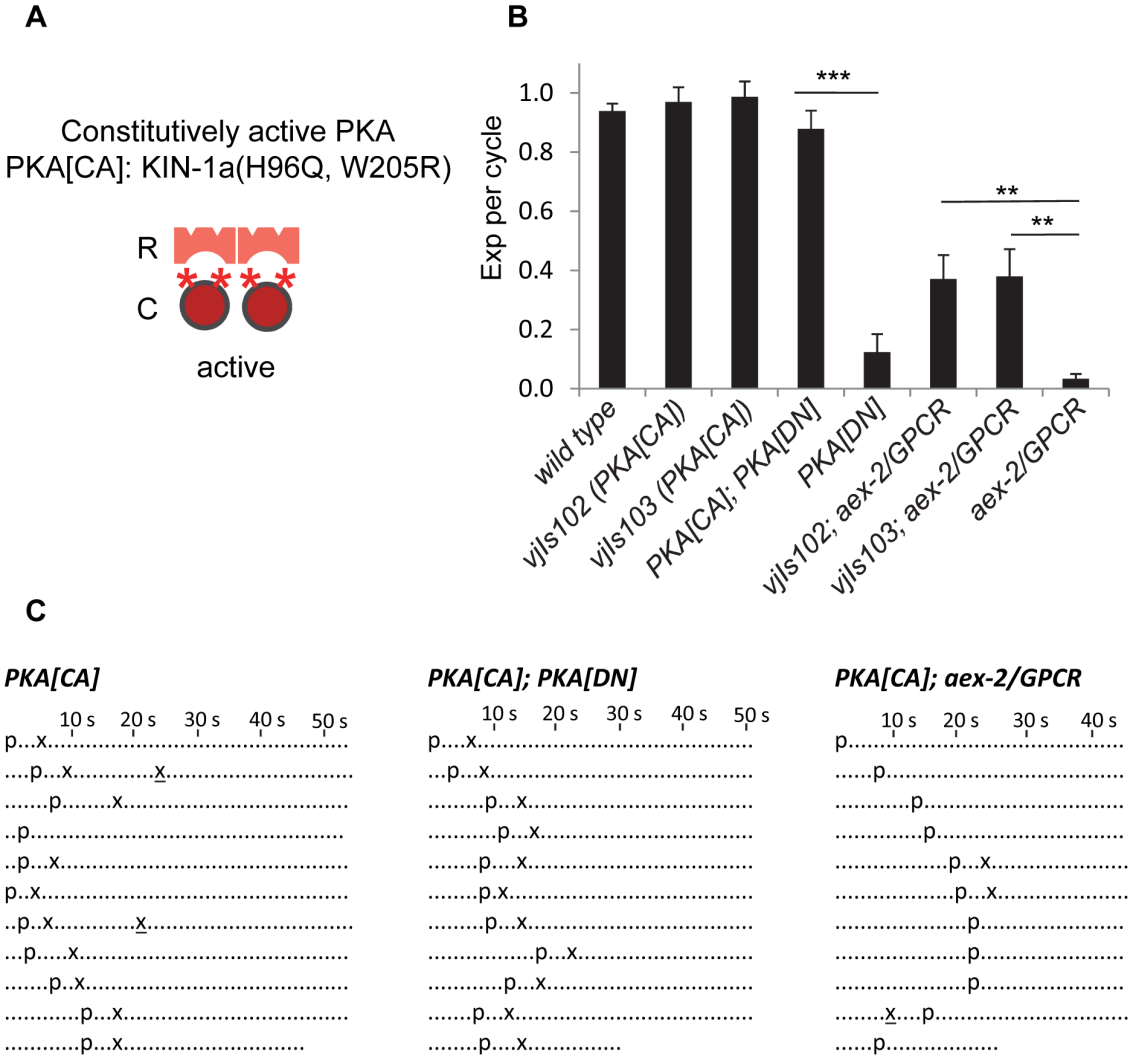
Constitutively active PKA in GABAergic neurons partially bypasses the requirement of AEX-2/GPCR. (A) Diagram showing the construction of the constitutively active PKA (PKA[CA]). “R” and “C” indicate the PKA regulatory and catalytic subunit, respectively. The two asterisks (*) represent the two mutations (H96Q, W205R) in the PKA catalytic subunit KIN-1a, which presumably disrupt its association with the regulatory subunit but do not affect its enzymatic activity. (B) Quantification of the Exp step of young adults with the indicated genotypes. PKA[CA] denotes PKA constitutively active transgenic worms (*vjIs102* and *vjIs103*) expressing the mutated catalytic subunit *kin-1a*(H96Q, W205R) in the GABAergic neurons using the *unc-47* full length promoter. *vjIs103* and *vjIs77* were used for the PKA[CA];PKA[DN] strain. (C) Representative ethograms of ten consecutive defecation cycles of young adult worms with the indicated genotypes. Each dot represents 1 s. “p” stands for the pBoc step and “x” indicates the Exp step. Ectopic Exp steps are indicated by “x”. Means and standard errors are shown. Asterisks indicate significant differences between indicated groups: ** P<0.01, *** P<0.005 in Student's t-test.

To independently confirm that PKA functions in the AEX-2/GPCR peptidergic signaling pathway to regulate the Exp step, we generated a constitutively active *kin-1*/PKA variant (referred to as PKA[CA]) and examined whether PKA[CA] transgenes could bypass the requirement of *nlp-40* or *aex-2*/GPCR. The His87 and Trp196 residues in the mouse PKA catalytic α subunit lie at the interface between the R and C subunits and are necessary for the interaction between them [28]. H87Q, W196R substitutions disrupt this interaction resulting in a constitutively active catalytic subunit that increases PKA activity in vivo [29], [30]. The *C. elegans* KIN-1a isoform shares 91% amino acid identity with the mouse PKA catalytic α subunit (Figure S2). We made the corresponding substitutions in the KIN-1a isoform (H96Q, W205R) (Figure 3A and S2), and generated two independent transgenic lines (*vjIs102* and *vjIs103*) expressing KIN-1a(H96Q, W205R) specifically in GABAergic neurons (using the full length *unc-47* promoter).

Several pieces of evidence support the notion that PKA[CA] transgenes confer constitutive PKA activity. First, animals expressing PKA[CA] transgenes had grossly normal defecation motor programs (Figure 3B and 3C) but occasionally displayed “ectopic” Exp following normal Exp steps (4 out of 100 cycles in *vjIs102* and 10 out of 100 cycles *vjIs103*. p = 0.11 and 0.03, respectively, n = 10, one tail t-test, compared to wild type) (Figure 3C). Ectopic Exp steps were also observed in *kin-2(ce179)* mutants (5 out of 100 cycles, p = 0.09, n = 10), which carry a mutation in the PKA regulatory subunit that is predicted to increase PKA catalytic activity [31]. Second, ectopic Exp steps are also observed in *gsa-1/*Gαs gain-of-function mutants, which mimic constitutive peptidergic activation of the pathway in GABAergic neurons [5]. Finally, PKA[CA] transgenes completely suppressed the Exp defects caused by PKA[DN] expression (Figure 3B and 3C), demonstrating that PKA[CA] is not inhibited by the PKA regulatory subunit.

We found that PKA[CA] transgenes partially restored Exp to *aex-2*/GPCR and *nlp-40* mutants (Figure 3B, 3C, and S3). Similarly, *kin-2* loss-of-function mutations restored Exp to *nlp-40* mutants to a similar extent as PKA[CA] transgenes (Figure S3). Constitutively active *acy-1*/adenylyl cyclase transgenes in GABAergic neurons have also been reported to partially rescue the Exp defects of *aex-2*/GPCR mutants [5]. Taken together, these results are consistent with the idea that cAMP generated by the NLP-40-AEX-2/GPCR peptidergic signaling pathway in the GABAergic neurons activates PKA, which drives Exp by promoting GABA release.

### PKA regulates rhythmic calcium influx in the DVB neuron

**Figure 4:**
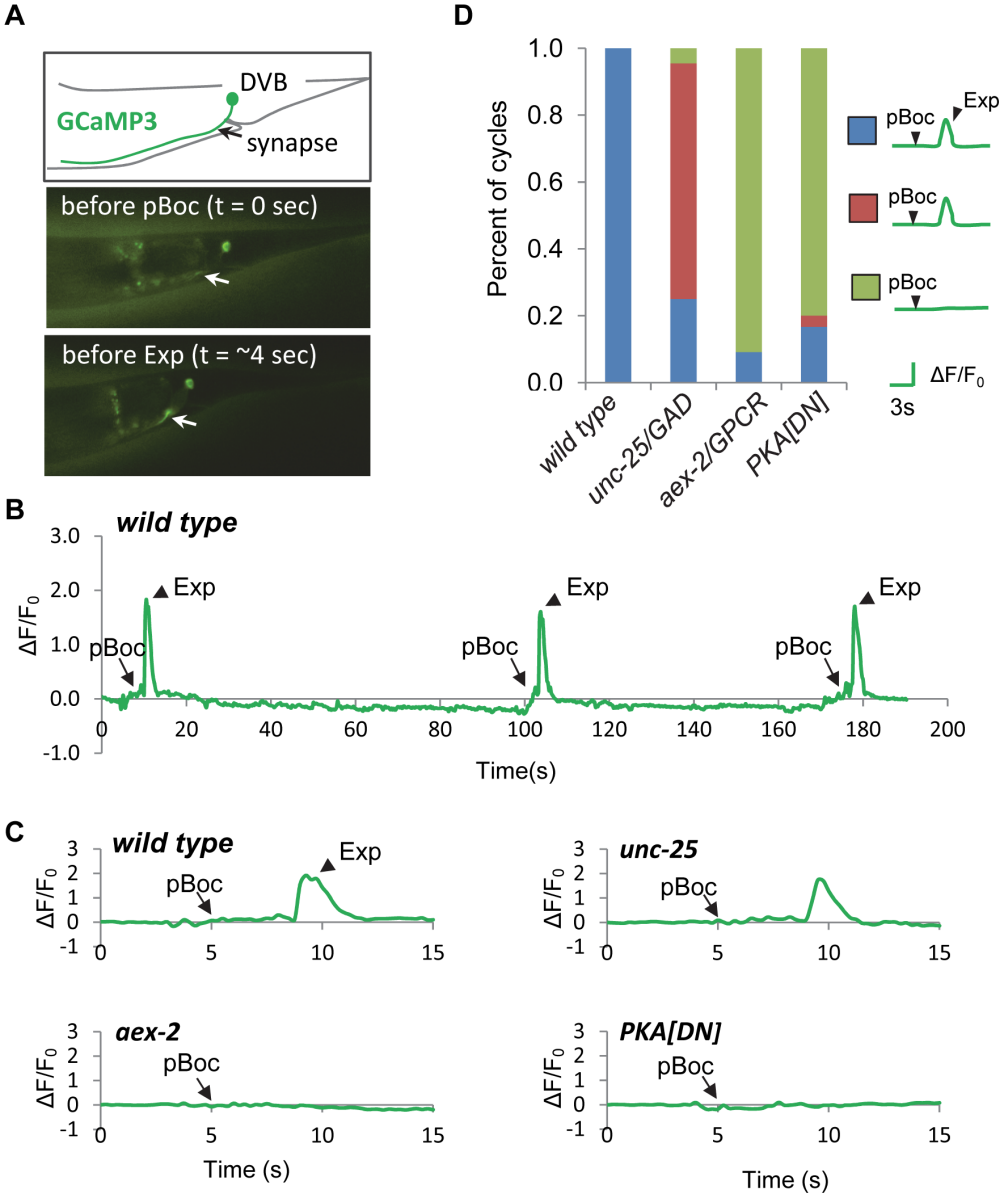
PKA is necessary for calcium influx in DVB neurons. (A) Expression of the genetically-encoded calcium indicator, GCaMP3 in DVB neurons (*vjIs58*). Top: diagram of the DVB neuron in the tail region. Synapse means the neuromuscular junction where the DVB neuron innervates enteric muscles. Middle and Bottom: two snapshots from a real-time imaging video of wild type animals showing an increase in fluorescence in synaptic region of DVB neurons, as indicated by the white arrow, right before the Exp step. (B) A representative trace of the GCaMP3 fluorescence in the synaptic region of DVB neurons in wild type animals showing DVB neurons are rhythmically activated during three consecutive defecation cycles. Note that the cycle length is longer than 50 seconds, likely due to *unc-13(s69)* mutation, which was used to immobilize the worms for calcium imaging (See Materials and Methods for details). (C) Representative traces of the GCaMP3 fluorescence in the synaptic region of DVB neurons in worms with the indicated genotypes. The observed pBoc step and Exp step are indicated by arrows and arrowheads, respectively. (D) Classification of the different patterns of pBoc, fluorescent spikes in DVB and Exp in worms with the indicated genotypes. *vjIs76* was used for the PKA[DN] strain. In (D), wild type: 23 cycles in 11 animals; *unc-25*: 44 cycles in 11 animals; *aex-2*: 44 cycles in 11 animals; PKA[DN]: 30 cycles in 12 animals.

We previously found that during the defecation motor program, the axons of DVB neurons display robust, rapid calcium transients that peak just before each Exp step, suggesting that rhythmic presynaptic calcium influx in DVB drives rhythmic GABA release from DVB neurons. Using a genetically-encoded calcium indicator, GCaMP3 [32], we found that fluorescent spikes in DVB axons began about 3 seconds following the pBoc step, reached maximal intensity about 1 second later (immediately before each Exp step), and returned to baseline within 2 seconds (Figure 4A, 4B and 4C and Video S1 and [23]). In most cases, calcium transients appeared to initiate in the synaptic region of DVB neurons and often would spread along the axon to the cell body. In wild type animals the “normal” pattern of pBoc-calcium transient-Exp was highly reproducible, occurring 100% of the time (23 cycles, 11 animals, Figure 4C and 4D). In mutants lacking *aex-2/*GPCR, only about 9% of cycles adopted a normal pattern (4 out of 44 cycles, 11 animals), and the remaining 91% of cycles lacked both the calcium transients and Exp (40 out of 44 cycles, 11 animals, Figure 4C and 4D and Video S2). In contrast, in *unc-25* mutants, which lack the GABA biosynthetic enzyme glutamic acid decarboxylase (GAD) [33], most of the cycles without Exp steps still produced a calcium spike (31 out of 33 cycles without Exp, 11 animals, Figure 4C and 4D and Video S3). These results show that the generation of calcium transients is dependent on the peptidergic signaling pathway but occurs independently of GABA release from DVB neurons, and are consistent with the idea that the calcium spikes in the synaptic region of DVB neurons drive the Exp step by triggering GABA release.

**Figure 5:**
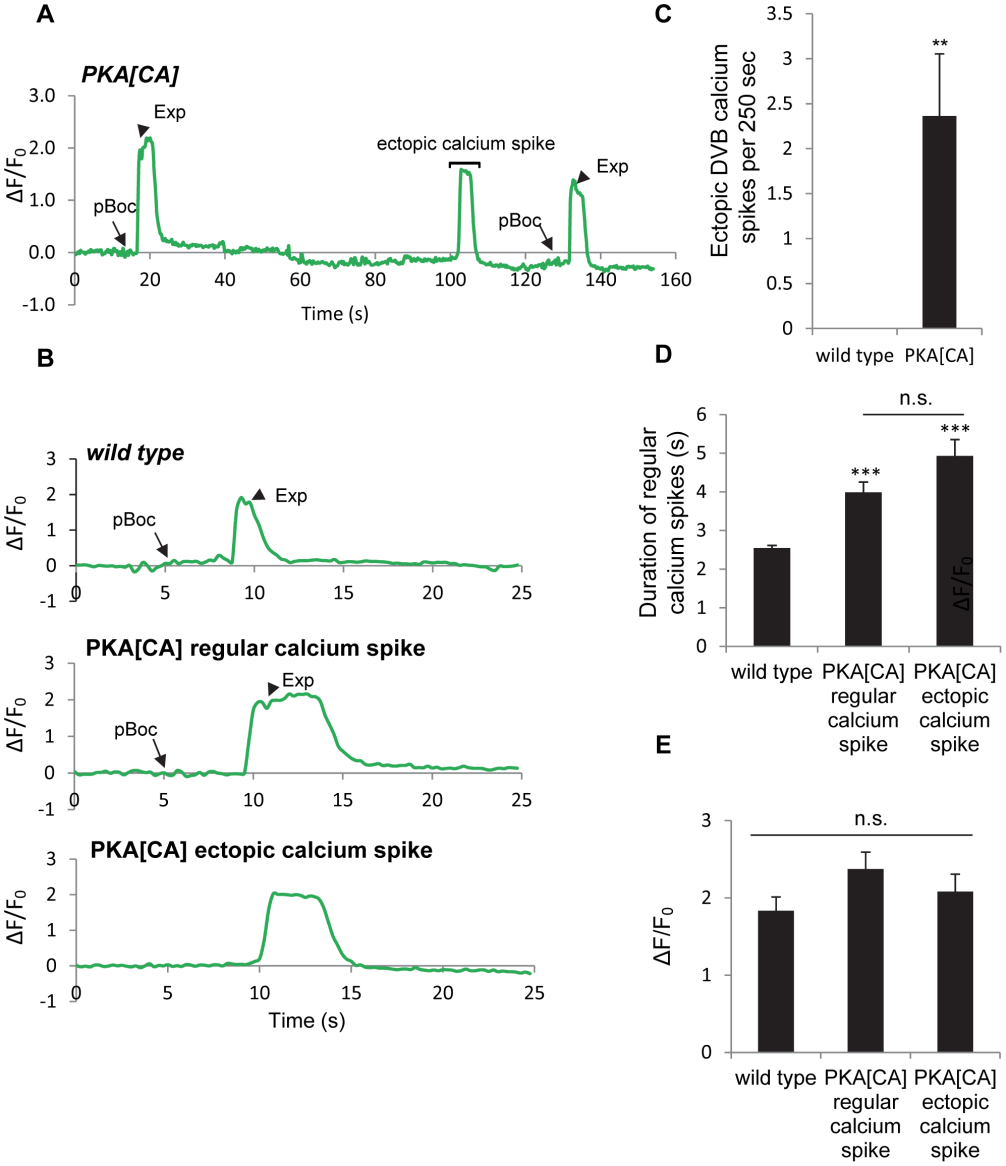
Constitutively active PKA causes ectopic calcium spikes in DVB neurons and increases calcium spike duration. (A) A representative trace of the GCaMP3 fluorescence in the synaptic region of DVB neurons in transgenic worms expressing PKA[CA] (*vjIs102*) during two consecutive defecation cycles. An ectopic calcium spike in DVB neurons was observed, as indicated by the bracket. Note that the cycle length is longer than 50 seconds, likely due to *unc-13(s69)* mutation, which was used to immobilize the worms for calcium imaging (See Materials and Methods for details). (B) Representative traces of the GCaMP3 fluorescence in the synaptic region of DVB neurons in worms with the indicated genotypes. The observed pBoc step and Exp step are indicated by arrows and arrowheads, respectively. (C) Average frequency of ectopic calcium spikes in DVB neurons during a 250-second imaging period in wild type animals (0.0±0.00 events per 250 seconds, n = 10 animals) and *vjIs102* (2.4±0.69 events per 250 seconds, n = 11 animals). (D) and (E) the duration and amplitude of regular DVB calcium spikes and ectopic calcium spikes in wild type and PKA[CA] (*vjIs102*) animals. Means and standard errors are shown. Asterisks indicate significant differences from wild type: ** P<0.01, *** P<0.005 in Student's t-test. “n.s.” indicates no significant difference between indicated groups.

Our behavioral results show that PKA activity may be necessary and sufficient for GABA release from the GABAergic neurons. In principal, PKA could control GABA release directly by acting on the synaptic vesicle machinery at presynaptic terminals or indirectly by regulating the excitability of the GABAergic neurons. To distinguish between these two possibilities, we examined whether PKA[DN] or PKA[CA] transgenes impacted GCaMP3 fluorescence spikes in DVB neurons during the defecation motor program. PKA[DN] transgenes produced a calcium pattern that was similar to that observed in *aex-2/*GPCR mutants: 80% of cycles lacked both calcium transients and the Exp steps (31 cycles, 12 animals, Figure 4C and 4D and Video S4). On the other hand, PKA[CA] transgenes caused “ectopic” fluorescent transients in DVB neurons, which occurred once or twice in between cycles on average (22 regular calcium spikes and 26 ectopic calcium spikes observed during 24 cycles, 11 animals, Figure 5A, 5B and 5C and Video S6). These “ectopic” fluorescent transients were occasionally associated with an ectopic Exp (6 out of the 26 ectopic calcium spikes, 11 animals, see Discussion). The duration of both the “normal” and “ectopic” fluorescent transients in PKA[CA] transgenic animals was significantly longer compared to wild type controls (Figure 5D and Video S5 and S6). However, the amplitude of calcium transients was not significantly different (Figure 5E, wild type: ΔF/F_0_ = 1.83±0.17, n = 30; PKA[CA]: normal calcium transients ΔF/F_0_ = 2.37±0.22, n = 21, p = 0.06; ectopic calcium transients, ΔF/F_0_ = 2.08±0.22, n = 26, p = 0.39, two tail t-test, compared to wild type). Taken together, we conclude that PKA is essential for the generation of calcium transients in DVB neurons during the defecation cycle. In addition, PKA activity is sufficient to generate calcium transients and may positively regulate the duration but not the amplitude of calcium transients.

### UNC-2/VGCC acts downstream of PKA and mediates calcium influx in the DVB neuron

**Figure 6:**
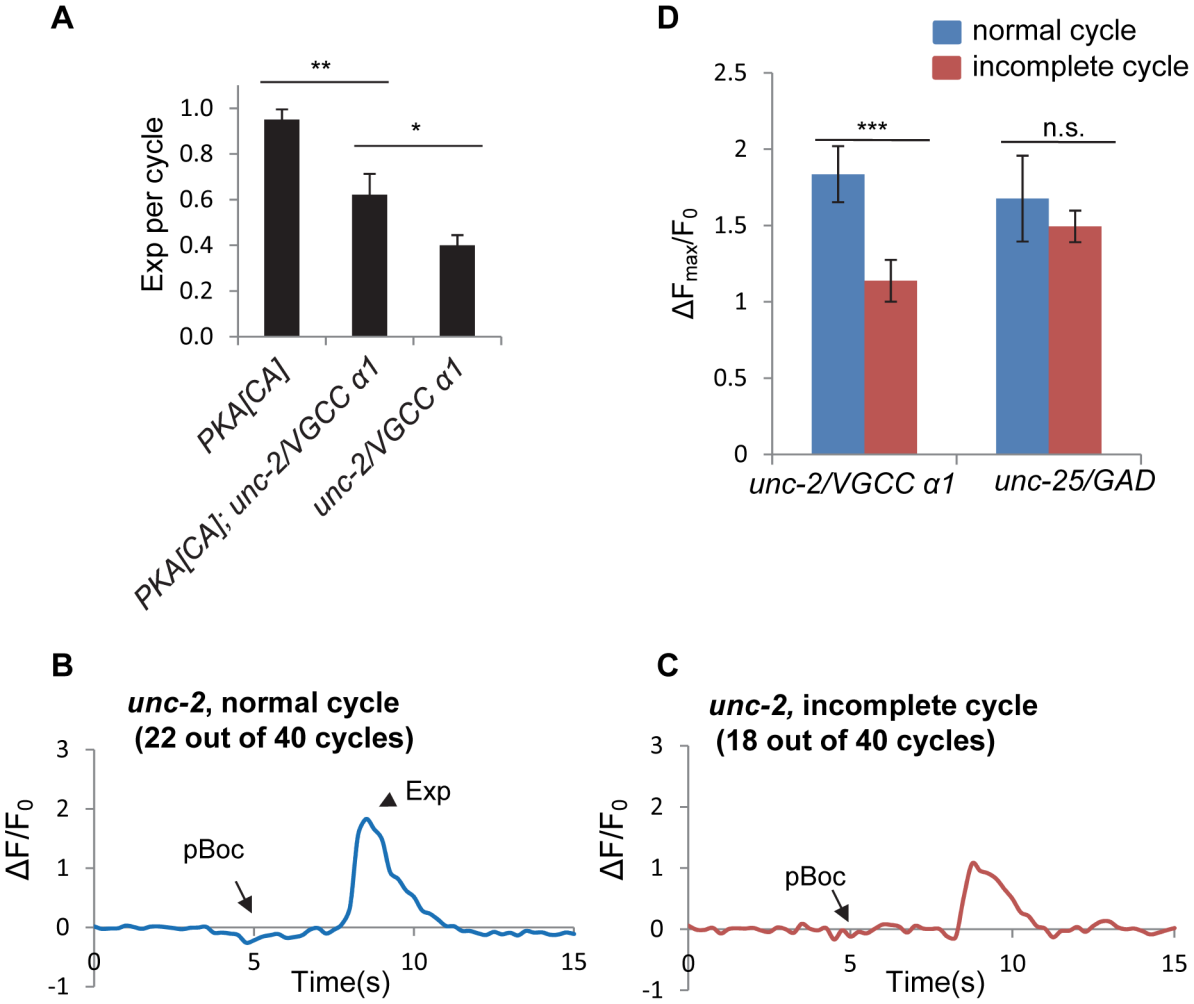
*unc-2*/VGCC functions downstream of PKA to mediate calcium influx in the GABAergic neurons. (A) Quantification of the Exp step of young adult worms with the indicated genotypes. *vjIs103* was used for the PKA[CA] strain. (B) and (C) Representative trace of GCaMP3 fluorescence in the synaptic region of DVB neurons in *unc-2* mutants in normal cycles, with the Exp step (B) and incomplete cycles, without the Exp step (C). (D) Comparison of the maximal change (ΔF_max_/F_0_) of GCaMP3 fluorescence in the synaptic region of DVB neurons between the normal cycles and the incomplete cycles in *unc-2* mutants and *unc-25* mutants. *vjIs58*, the transgenic strain with GCaMP3 expressed in the DVB neuron, was used. Normal cycles: n = 22 in *unc-2(lj1)* mutants; n = 10 in *unc-25(e156)* mutants. Incomplete cycles: n = 18 in *unc-2(lj1)* mutants; n = 31 in *unc-25(e156)* mutants. Means and standard errors are shown. Asterisks indicate significant differences: * P<0.05, *** P<0.0005 in Student's t-test.

Voltage-gated calcium channels (VGCCs) are critical for presynaptic calcium influx during regulated neurotransmitter release [34]. *unc-2* encodes the α1 subunit of the *C. elegans* P/Q type VGCC. UNC-2 localizes to presynaptic terminals, and promotes calcium influx and neurotransmitter release [35], [36]. *unc-2* has been reported to be expressed in the DVB neuron, and to regulate the Exp step [37]. Consistently, we observed the Exp step in only about 40% of the defecation cycles in *unc-2(lj1)* null mutants (Figure 6A). In *unc-2* mutants expressing PKA[CA], the Exp step was observed in about 60% of cycles (Figure 6A). The incomplete restoration of the Exp step by PKA[CA] is consistent with the idea that UNC-2 functions downstream of or in parallel to PKA to regulate Exp.

To test whether the rhythmic calcium transients are mediated by UNC-2, we examined GCaMP3 fluorescence in synaptic regions of DVB neurons in *unc-2* mutants. Surprisingly, fluorescent transients were observed in all cycles including the normal cycles that had the Exp steps and the incomplete cycles without the Exp steps (Figure 6B and 6C and Video S7 and S8). However, there was a significant reduction in the average amplitude of fluorescent transients in the incomplete cycles (that lacked Exp, n = 18) compared to the fluorescent transients in the normal cycles (those with Exp, n = 22) (Figure 6B, 6C and 6D). To determine if the reduction in fluorescence amplitude in incomplete cycles was a secondary effect of compromised Exp in these mutants, we examined fluorescence amplitudes in mutants lacking *unc-25/*GAD. We found no difference in the average amplitude between normal and incomplete cycles in *unc-25/*GAD mutants (n = 10 and 31 cycles, respectively, and Figure 6D). These results indicate that UNC-2 is not absolutely required for the generation of calcium transients but rather regulates the size of calcium transients, possibly allowing them to reach the threshold required for triggering Exp.

### Other non voltage-gated calcium channels may also mediate calcium influx in the DVB neuron

**Figure 7:**
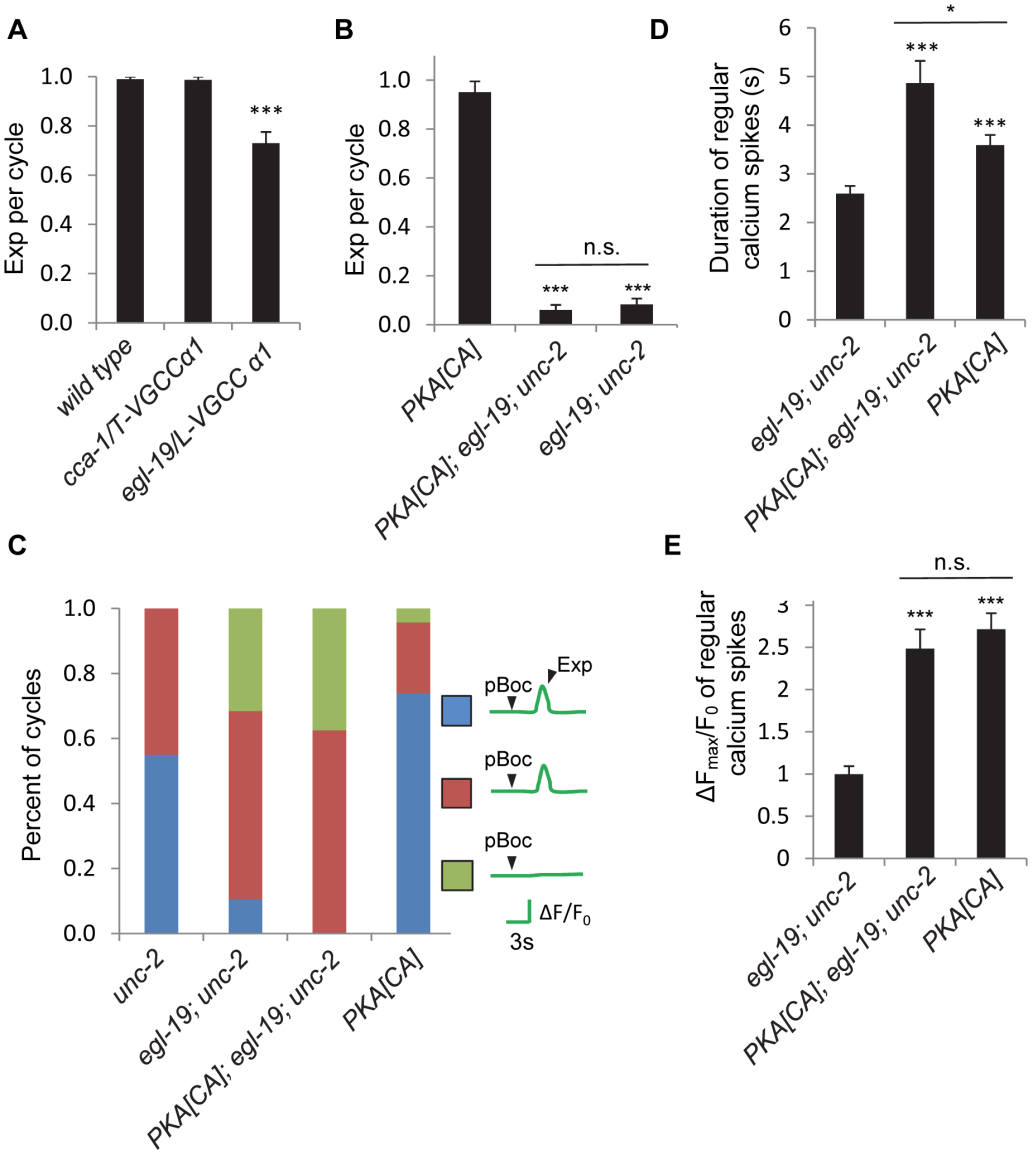
Other non voltage-gated calcium channels are required from calcium influx in the GABAergic neurons. (A) and (B) Quantification of the Exp step of young adult worms with the indicated genotype types. (C) Classification of different patterns of pBoc, fluorescent spikes in DVB and Exp in worms with the indicated genotypes. *unc-2*: 40 cycles in 21 animals, *egl-19; unc-2*: 19 cycles in 11 animals, *PKA[CA]; egl-19; unc-2*: 24 cycles in 12 animals, PKA[CA]: 23 cycles in 12 animals. (D) and (E) Quantification of the duration and amplitude of regular DVB calcium spikes in worms with indicated genotypes. PKA[CA] represents transgenic worms with constitutively active PKA specifically expressed in GABAergic neurons (*vjIs103* in (B) and *vjIs102* in (C, D)). Note that in (C), (D) and (E), *unc-2* and PKA[CA] strains, but not the *egl-19; unc-2* and the PKA[CA]; *egl-19; unc-2* strains contain the *unc-13(s69)* mutation for immobilization, as *egl-19;unc-2* alone were almost completely paralyzed. *vjIs58*, the transgenic strain with GCaMP3 expressed in the DVB neuron, was used for the *unc-2* strain; while *vjIs64* was used for calcium imaging in other genotypes. Means and standard errors are shown. Asterisks indicate significant difference between indicated group and significant difference from wild type in (A), PKA[CA] in (B) and *egl-19; unc-2 mutants* in (D) and (E): * p<0.05; ***P<0.005 in Student's t-test. “n.s.” indicates no significant difference between indicated groups.

Because *unc-2* does not appear be to the only channel to mediate the calcium transients in DVB neurons for the Exp step, we next sought to identify other calcium channels that might function in this process. The *C. elegans* genome encodes two additional VGCC α1 subunits: *egl-19*, the L-type α1 subunit, and *cca-1*, the T-type α1 subunit [38], [39]. We found that loss-of-function *cca-1/*VGCC mutants had normal Exp steps, whereas loss-of-function *egl-19/*VGCC mutants displayed a modest reduction in the number of cycles with Exp (Figure 7A). Interestingly, in double mutants lacking both *unc-2* and *egl-19*, the Exp step was nearly eliminated (Figure 7B). Furthermore, *PKA[CA]; egl-19; unc-2* triple mutants had the same, severely reduced Exp frequency as the *egl-19; unc-2* double mutants (Figure 7B). Thus, two VGCCs, *egl-19* and *unc-2*, function downstream of PKA to control the Exp step.

To directly test whether *egl-19/*VGCC mediates calcium influx in DVB neurons, we next examined the effects of *egl-19* mutations on GCaMP3 fluorescence in DVB neurons. We were unable to analyze calcium influx in *egl-19* mutants due to an unexpected genetic interaction between *egl-19* and *unc-13(s69)* (used to paralyze animals for imaging without stopping the defecation cycle) that suppressed the Exp defects of *egl-19* mutants (see Materials and Methods). Instead, we examined calcium influx in *egl-19; unc-2* double mutants (which were sufficiently paralyzed for imaging without the *unc-13(s69)* mutation). We found that the fraction of incomplete cycles increased to 80% (from 40% in *unc-2* mutants, Figure 7C and Video S9). Finally, no calcium influx was observed at all in 35% of the incomplete cycles, a defect that was not observed in the *unc-2* mutants alone (Figure 7C and Video S10). In *egl-19; unc-2* double mutants expressing PKA[CA] transgenes, these defects in calcium influx were as severe as those observed in *egl-19; unc-2* mutants (Figure 7C), suggesting that *egl-19* and *unc-2* function downstream of PKA to promote calcium influx into DVB.

*egl-19* and *unc-2* mutations were not able to block all effects of PKA[CA] transgenes, including the increased frequency of ectopic calcium transients and the increased duration of normal and ectopic calcium transients (Figures 7D and S4 and Video S11 and S12). In addition, the amplitude of regular calcium spikes of the incomplete cycles *in egl-19; unc-2* mutants expressing PKA[CA] transgenic was similar to those in PKA[CA] transgenic animals, but significantly larger than those observe in *egl-19; unc-2* double mutants (Figure 7E). Together these results suggest that the effects of *egl-19* and *unc-2* mutations on Exp can in part be attributed to their requirement for calcium influx in DVB neurons and that additional calcium channels must act downstream of PKA to mediate calcium influx in DVB.

## Discussion

In this study, we identify PKA as the major downstream target of cAMP in the NLP-40-AEX-2/GPCR peptidergic signaling pathway that functions in the GABAergic neurons to regulate the Exp step during the defecation motor program in *C. elegans*. PKA controls rhythmic activation of the GABAergic neurons by promoting presynaptic calcium influx in these neurons. We find that the mechanism by which PKA functions to promote rhythmic calcium influx is partially dependent on the P/Q-type VGCC, UNC-2 and the L-type VGCC, EGL-19. These results suggest that PKA promotes rhythmic neurotransmitter release by controlling calcium influx in neurons during a rhythmic behavior.

### PKA is essential for presynaptic calcium influx in DVB neurons

Our results suggest that PKA functions as an essential cue for calcium influx in neurons for their activation to control rhythmic behaviors, because both calcium spikes in DVB neurons and Exp steps are abolished in most of the defecation cycles in animals expressing PKA[DN] transgenes (Figure 2B, 2D, 4C and 4D). To our knowledge, this is the first in vivo example showing that PKA in neurons is absolutely required for the execution of a rhythmic behavior. In many preparations, PKA has been shown to modulate biological processes by regulating intracellular calcium concentration. One classic example is that PKA mediates the enhancement of calcium influx in cardiac myocytes to modulate the rate of heart beating in response to norepinephrine from the sympathetic nervous system [8]. In neurons, PKA also has a modulatory role in regulation of synaptic transmission and synaptic plasticity by phosphorylating several synaptic vesicle proteins that function downstream of calcium influx [13].

### UNC-2 and EGL-19 mediate part of PKA-dependent calcium influx in DVB neurons

How does PKA control calcium influx in the GABAergic neurons? Our results suggest that PKA acts at least partially through UNC-2, the P/Q-type VGCC, and EGL-19, the L-type VGCC. *unc-2* has been reported to be expressed in the DVB neuron and *egl-19* is expressed in many neurons as well [37], [38], . Our behavioral and calcium imaging analysis of *egl-19; unc-2* double mutants expressing PKA[CA] transgenes suggests that one or both of these channels must also function in enteric muscles to promote Exp since PKA[CA] can restore normal calcium spike amplitudes but it fails to restore the Exp step in *egl-19; unc-2* double mutants (Figure 7B, 7C and 7E). Consistent with this idea, *egl-19* has been reported to be expressed in muscles, including some of the enteric muscles [38]. Our data indicates that other non voltage-gated calcium channels must be also required for calcium influx in DVB neurons for the Exp step, since *egl-19 and unc-2* mutations together could not completely block the calcium influx in DVB neurons or the effects of PKA[CA] transgenes on calcium influx (Figure 7C, 7D and 7E). The identification of these channels will further our understanding of the mechanism underlying PKA-dependent calcium influx in these neurons.

PKA may regulate calcium influx either by direct phosphorylation of UNC-2 and/or EGL-19 or by an indirect mechanism involving, for example, the regulation of membrane potential. Indeed, both mechanisms have been reported. The fight-or-flight response is controlled by PKA-dependent phosphorylation of the L-type calcium channels Ca_v_1.1 and Ca_v_1.2 which mediate calcium influx in skeletal and cardiac muscles, respectively, to enhance their contraction [8], [41]. EGL-19 does not have the homologous phosphorylation site. In pancreatic beta cells, PKA has been reported to phosphorylate ATP-sensitive potassium channels to regulate the membrane potential [42]. Interestingly, the *egl-36*/Shaw type potassium channel functions in DVB to regulate the Exp step [43], raising the possibility that it may be a target for PKA.

Our results also show that PKA[CA] transgenes increases the duration of calcium spikes in DVB neurons in both wild type and *egl-19; unc-2* mutants, suggesting that PKA regulates calcium spike dynamics independently of *egl-19* and *unc-2*. PKA might regulate the open time or the inactivation of other calcium channels or reduce the rate of calcium clearance from the synaptic region in DVB neurons. Consistent with our observation, injection of the PKA catalytic subunits in cells has been reported to increase the duration of calcium currents [44]–[46].

### The activity of PKA in the GABAergic neurons during the defecation cycle

Our previous work indicates that NLP-40 functions as the timing signal from the intestine and it delivers the timing information to the GABAergic neurons by instructing their rhythmic activation [23]. In this study, we show that the downstream effector PKA is also instructive for the activation of the GABAergic neurons, because the PKA[CA] transgenes can elicit ectopic calcium spikes in between cycles. The observation that ectopic calcium spikes in DVB neurons do not occur in wild type animals suggests that endogenous PKA activity in these neurons must somehow be turned down rapidly following each Exp step. Thus, we propose a model in which rhythmic activation of PKA by the NLP-40-AEX-2/GPCR peptidergic pathway stimulates rhythmic calcium influx in the GABAergic neurons to drive the Exp step. It will be interesting to directly determine whether cAMP levels and/or PKA activity oscillate in DVB neurons and whether these oscillations correspond with the calcium oscillations in vivo. Several negative feedback mechanisms following PKA activation that have been reported in other preparations may also help establish rhythmic PKA activity in DVB neurons. These include the activation of by phosphodiesterases (PDEs) that break down cAMP, calcium-mediated inhibition of adenylyl cyclases and activation of specific phosphatases that counteract PKA activity [9], [47], [48]. PKA activity has been reported to be essential for the initiation of the cAMP-PKA-Ca^2+^ oscillation circuit in insulin-secreting MIN6 beta cells (Ni et al., 2010). Thus, the interplay between cAMP, PKA and calcium may be a general mechanism by which PKA generates oscillatory signaling circuits in neurons to control rhythmic behaviors.

Rhythmic PKA activation may be essential for the reliability of the Exp step during the defecation cycle, because PKA[CA] transgenes cannot fully restore Exp to animals that lack the NLP-40-AEX-2/GPCR peptidergic signaling pathway components (Figure 3B). Similarly, constitutive *acy-1*/adenylyl cyclase expression in the GABAergic neurons also only partially rescues the Exp defects of *aex-2* mutants [5]. Interestingly, *gsa-1(gf)/*Gαs mutations almost fully rescue the Exp defects in *aex-2/*GPCR mutants [5]. Since GSA-1/Gαs functions upstream of both adenylyl cyclase and PKA, it is possible that *gsa-1(gf)* mutants retain the proper negative feedback mechanisms that allow PKA activity to remain rhythmic.

Rhythmic PKA activation may work together with other mechanisms to ensure the proper execution of the Exp step. Indeed, it has been postulated that a permissive signal may control the refractory period of enteric muscles by allowing Exp to occur only within a small window of time following the beginning of each cycle (the pBoc step) [5], [23]. We speculate that this permissive signal may be entrained to the pacemaker activity in the intestine, because most of the Exp steps observed in the *aex-2* mutants expressing PKA[CA] did not happen at random times, but rather occurred a few seconds after the pBoc step. The absence of this permissive signal in between cycles may also explain why PKA[CA] elicits ectopic calcium spikes in DVB neurons more frequently than ectopic Exp steps. The identification of this permissive signal or the nature of the refractory period of enteric muscles would provide more comprehensive understanding on how the rhythmic Exp is reliably generated.

### Using genetically-encoded PKA transgenes to dissect the function of PKA in vivo

In mammals, several genes encode different PKA regulatory and catalytic subunits, and each of these genes may have several different isoforms due to alternative splicing [12]. Although many PKA isoforms have been knocked out and knocked in in mice to dissect the role of PKA in vivo, compensation by remaining isoforms has complicated the interpretation of these studies [27], [49], [50]. Unlike mammals, *C. elegans* has a single gene (*kin-2*) for the PKA regulatory subunit and a single gene (*kin-1*) for the catalytic subunit, both of which share high similarities with their counterparts in higher mammals. Thus, *C. elegans* has the potential to be a good genetic model to dissect the physiological roles of PKA. However, studies on the contribution of PKA signaling in *C. elegans* have been limited since null mutants of either *kin-1/*catalytic subunit or *kin-2/*regulatory subunit are lethal. Various strategies using weak alleles of *kin-2*, pharmacological treatments with PKA inhibitors, and RNAi-mediated knockdown, have revealed roles for PKA in regulating neurotransmitter release, axon regeneration and behaviors [2], [31], [51]. However, non-specific effects of pharmacological PKA inhibitors and spreading of RNAi limit the utility of these approaches [52], [53]. The ability to manipulate PKA activity in a tissue specific manner using the PKA variants developed in this study represents a powerful approach for probing the function of PKA signaling in vivo.

## Materials and Methods

### Strains

Strains were maintained at 20°C on NGM plates with *E. coli* strain OP50 as food. The Bristol strain N2 was used as reference strain. The strains used in this study: OJ1672 *cng-2(tm4267) IV*, FX05036 *cng-4/che-6 (tm5036) IV*, OJ1748 *tax-2(p671) I*, KJ5562 *tax-4(p678) III; cng-3(jh113) IV; cng-1(jh111) V*, RB830 *epac-1(ok655) III*, DG3393 *tnEx109; kin-1(ok338) I*, OJ1540 *aex-2(sa3) X*, OJ1603 *vjIs76 [Pttx-3::RFP*, *40 ng/µl; Punc-47(FL):: kin-2a(G310D)*, *50 ng/µl] V*, OJ1601 *vjIs77 [Pttx-3::RFP*, *40 ng/µl; Punc-47(FL):: kin-2a(G310D)*, *50 ng/µl] IV*, KG421 *gsa-1(ce81) I*, OJ1908 *gsa-1(ce81) I; vjIs77 IV*, OJ1854 *vjIs102 [Pmyo-2::NLS::GFP*, *10 ng/µl; Punc-47(FL)::kin-1a(H96R*, *W205Q)*, *50 ng/µl] V*, OJ1858 *vjIs103 [Pmyo-2::NLS::GFP*, *10 ng/µl; Punc-47(FL)::kin-1a(H96R*, *W205Q*, *50 ng/µl)] I*, OJ1896 *vjIs103 I; vjIs77 IV*, OJ1909 *vjIs102 V; aex-2(sa3) X*, OJ1910 *vjIs103 I; aex-2(sa3) X*, OJ680 *unc-13(s69) I*, OJ1213 *vjIs58 [Pmyo-2::NLS::mCherry*, *10 ng/µl; Punc-47(mini)::GCaMP3*, *125 ng/µl] IV*, OJ1443 *unc-13(s69) I; vjIS58 IV*, OJ1468 *unc-13(s69) I; vjIS58 IV; aex-2(sa3) X*, OJ1759 *unc-13(s69) I; vjIs58 IV; vjIs76 V*, OJ1859 *unc-13(s69) I; unc-25(e156) III; vjIs58 IV*, OJ1917 *unc-13(s69) I; vjIs58 IV; vjIs102 V*, OJ1526 *unc-2(lj1) X*; OJ1899 *vjIs103 I; unc-2(lj1) X*; OJ1919 *unc-13(s69) I; vjIs58 IV; unc-2(lj1) X*, JD21 *cca-1(ad1650) X*, OJ1911 *egl-19(n582) IV*, OJ1925 *egl-19(n582) IV; unc-2(lj1) X*, OJ1924 *vjIs103 I; egl-19(n582) IV; unc-2(lj1) X*, OJ1351 *vjIs64 [Pmyo-2::NLS::mCherry*, *10 ng/µl; Punc-47(mini)::GCaMP3*, *125 ng/µl] II*, OJ1905 *unc-13(s69) I; vjIs64 II*, OJ1918 *vjIs64 II; egl-19(n582) IV; unc-2(lj1) X*, OJ1923 *unc-13(s69) I; vjIs64 II; vjIs102 V*, OJ1949 *vjIs64 II; egl-19(n582) IV; vjIs102 V; unc-2(lj1) X*, OJ794 *nlp-40(tm4085) I*, OJ1524 *kin-2(ce179) X*, OJ1525 *nlp-40(tm4085) I; kin-2(ce179) X*, OJ1855 *nlp-40(tm4085) I; vjIs102 V*, OJ1856 *nlp-40(tm4085) vjIs103 I*.

*cng-2(tm4267)* mutants contain a 330 bp deletion and a single nucleotide (g) insertion (www.wormbase.org), which is predicted to generate a frame shift that truncates CNG-2 at the C-terminus. *cng-2(tm4267)* was originally reported to be sterile and lethal (Mitani Lab). However, we found that after outcross *cng-2(tm4267)* mutants were viable.

### Behavioral assay for the defecation motor program

The defecation motor program was analyzed as previously described [19], [54]. Briefly, L4 stage hermaphrodites were transferred to a new plate. After about 20–24 hours, each worm was transferred to a fresh NMG plate and let to settle down for at least five minutes. Ten consecutive defecation cycles were scored for each worm using the Etho program software (http://depts.washington.edu/jtlab/software/otherSoftware.html) [54]. Only the pBoc and the Exp steps were scored, omitting the aBoc step. 8–10 worms were assayed for each genotype. “Exp per cycle” for each worm was calculated as the ratio of Exp over pBoc. The results were present as mean ± sem for each genotype. Unpaired two-tail Student's t-test with unequal variance was used to examine the significant difference between two different genotypes.

### Molecular biology

The backbone of the plasmids constructed below was pPD49.26 (A. Fire).

P*unc-47(FL)*, the 1444 bp full length *unc-47* promoter, which was expressed in all GABAergic neurons in *C. elegans* [5], was amplified from N2 genomic DNA using 5′ primer: ccccccGCATGCatgttgtcatcacttcaaactt and 3′ primer ccccccGGATCCctgtaatgaaataaatgtgacgctg. The PCR product was partially digested with SphI and BamHI, and cloned into the MCSI of a derivative plasmid of pPD49.26.

P*unc-47(mini)*, the 215 bp *unc-47* mini promoter, which was only expressed in a subset of GABAergic neurons (4 RME, RIS, AVL and DVB neurons) [55], was amplified from N2 genomic DNA with 5′ primer ccccccGCATGCCTGCAGctttcggtttggagagtag and 3′ primer ccccccGGATCCctgtaatgaaataaatgtgacgctg. The PCR product was digested with SphI and BamHI, and cloned into the MCSI of a derivative plasmid of pPD49.26 (with AsiSI and NotI inserted between the NheI and KpnI in MCS II).

GCaMP3 (1353 bp), the genetically-encoded calcium indicator [32], was cloned from a plasmid with GCaMP3 sequence (a gift from Robert Chow, USC) by PCR with 5′ primer ccccccGCGATCGCAAAAatgggttctcatcatcatcatc and 3′ primer ccccccGCGGCCGCttacttcgctgtcatcatttg. The PCR product was digested with AsiSI and NotI, and was cloned into a derivative of pPD49.26 that had *unc-47* mini promoter in MCS I and had AsiSI and NotI sites inserted between NheI and KpnI in MCS II to generate the plasmid pHW100: P*unc-47(mini)*::GCaMP3.

The wild type cDNA of *kin-2a* (1101 bp) was cloned from an N2 cDNA library (synthesized using NEB Protoscript RT-PCR kit) with 5′ primer ccccccGCTAGCAAAAatgtcgggtggaaacgaagag and 3′ primer ccccccGGTACCttaggtcatcagtttgacgtatgag. The Gly310 residue in KIN-2a was corresponding to the Gly324 residue in the site B of the mouse PKA regulatory subunit that will generate dominate negative PKA when it is mutated to Asp [26]. The *kin-2a* (G310D) variant was generated by two-step overlapping PCR using the following primers: pair 1 (5′ primer ccccccGCTAGCAAAAatgtcgggtggaaacgaagag and 3′ primer gaagaagagcgatttcGTcgaaatagtccgacattccaag and pair 2 (5′ primer cttggaatgtcggactatttcgACgaaatcgctcttcttc and 3′ primer ccccccGGTACCttaggtcatcagtttgacgtatgag).The final overlapping PCR product was digested with NheI and KpnI, and then cloned into the MCS II in a derivative plasmid from pPD49.26 that contained the *unc-47* full length promoter in MCS I to generate the plasmid pHW154: P*unc-47(FL)*::*kin-2a*(G310D).

The wild type cDNA of *kin-1*a (1080 bp) was cloned from N2 cDNA library with 5′ primer ccccccGCTAGCAAAAatgctcaagtttctgaaacc and 3′ primer ccccccGGTACCttaaaactcggcaaactctttg. The His96 and Trp205 residues in KIN-1a are corresponding to the His87 and Trp196 in the mouse PKA catalytic subunits which would generate constitutively active PKA when they are mutated to Gln and Arg, respectively [30]. The KIN-1a (H96R, W205Q) variant was created by two-step overlapping PCR using the following primers: pair 1(5′ primer ccccccgctagcAAAAatgctcaagtttctgaaacc and 3′ primer gaatgcgcttttcgttcaacgtTtgctccacttgcttgagttttac), pair 2(5′ primer gtaaaactcaagcaagtggagcaAacgttgaacgaaaagcgcattc and 3′ primer tctggtgtgccgcacaatgtccTcgttcgtcctttgacacgtttc) and pair 3(5′ primer gaaacgtgtcaaaggacgaacgAggacattgtgcggcacaccaga and 3′ primer ccccccGGTACCttaaaactcggcaaactctttg).The final overlapping PCR product was digested with NheI and KpnI, was cloned into the MCS II in a derivative plasmid from pPD49.26 that contained the *unc-47* full length promoter in MCS I to generate the plasmid pHW173: P*unc-47*(FL)::*kin-1a*(H96R, W205Q).

Sequencing was performed to confirm the mutations *kin-2a*(G310D) and *kin-1a*(H96R, W205Q) in the pHW154 and pHW173, respectively.

### Generation of transgenic animals

Microinjection of expression plasmids into the gonad of *C. elegans* was performed to generate transgenic animals with extrachromosomal arrays, according to the standard procedure [56]. Generally, total DNA concentration of the injection solution was 100 ng/µl (using the plasmid pBluescript to fill up if needed). The extrachromosomal arrays were integrated into the genome to generate stable transgenic worms using UV irradiation. The integrated transgenic lines were outcrossed at least 6 times.

The plasmid pHW154 (P*unc-47(FL)*::*kin-2a*(G310D)) was injected at 50 ng/µl, together with the co-injection marker plasmid KP708 (P*ttx-3*::RFP) at 40 ng/µl to generate the array *vjEx582* [P*ttx-3::RFP*, 40 ng/µl; P*unc-47(FL)*:: *kin-2a*(G310D), 50 ng/µl]. This array was integrated into genome to generate the PKA[DN] transgenic strains (*vjIs76* and *vjIs77*) with dominant negative PKA specifically expressed in GABAergic neurons.

The plasmid pHW173 (P*unc-47*(FL)::*kin-1a*(H96R, W205Q)) was injected at 50/µl, together with the co-injection maker KP1106 (P*myo-2*::NLS::GFP) at 10 ng/µl to generate the array *vjEx709* [P*myo-2*::NLS::mCherry, 10 ng/µl; P*unc-47(FL)*:: *kin-1a*(H96R, W205Q), 50 ng/µl]. This extrachomosomal array was integrated into genome to generate the PKA[CA] transgenic strains (*vjIs102* and *vjIs103*) with constitutively active PKA specifically expressed in GABAergic neurons.

The plasmid pHW100 (P*unc-47(mini)*::GCaMP3) was injected at 125 ng/µl, together with co-injection marker plasmid KP1368 (P*myo-2*::NLS::mCherry) at 10 ng/µl to generate the array *vjEx429* [P*myo-2*::NLS::mCherry,10 ng/µl; P*unc-47(mini)*::GCaMP3,125 ng/µl]. This array was integrated into genome to generate transgenic strains *vjIs58* and *vjIs64*, which expressed GCaMP3 in AVL and DVB neurons.

### In vivo calcium imaging

The calcium imaging experiment was performed as previously described [23]. We used two independent transgenic strains (*vjIs58* and *vjIs64*) with AVL and DVB neurons expressing GCaMP3 to perform in vivo calcium imaging on DVB neurons. Both strains had normal Exp steps (data not shown). Although *unc-13(s69)* mutants had a longer cycle length (78.5±11.4 s, mean ±SD, n = 8), they were also most completely paralyzed and still had normal Exp steps [23]. Thus, *unc-13(s69)* mutation was included in all strains to immobilize animals for calcium imaging, except for those strains with *elg-19(n582);unc-2(lj1)* double mutations, because the *elg-19(n582);unc-2(lj1)* double mutants were almost completely paralyzed. Young adult worms were transferred to NGM-agarose plate seeded with the food OP50. These agarose plates were topped with cover slides and imaged under a Nikon eclipse 90i microscope equipped with a Nikon Plan Apo 40× oil objective (N.A. = 1.0), a standard GFP filter and a Photometrics Coolsnap ES^2^ camera. Only those worms kept continuous pumping and positioned laterally with the left side pointing to the objective were selected for imaging. Time lapse imaging was obtained using Metamorph 7.0 software (Universal Imaging). Each worm was recorded for 250 s at 4 frames per second (3×3 binning, exposure time ranging from 5 ms to 80 ms dependent on the baseline GCaMP3 fluorescence in DVB neuron in each individual worm). Unlike DVB, AVL neuron is located in the head, so we could not routinely perform the calcium imaging on AVL and observe the Exp step in the same field. However, during some experiments, we did observe that AVL fired at the same time as DVB in coiled worms in which we could see AVL and Exp in the same field.

The quantification of the GCaMP3 fluorescence intensity in the synaptic region of DVB neurons using Metamorph 7.0 software (Universal Imaging) was performed as previously described [23]. The synaptic region of DVB neurons was manually selected as region of interest (ROI) and the average of the GCaMP3 fluorescence intensity for each frame was recorded. Meanwhile, a similar area near the tail region was used as background fluorescence for each frame. The GCaMP3 fluorescence (F) was defined as the (ROI - background). The average of the GCaMP3 fluorescence in the first 10 frames was used as baseline fluorescence F_0_. For each frame, the change of the GCaMP3 was presented as ΔF/F_0_ = (F-F_0_)/F_0_. The duration of each calcium spike was defined as the time differences between the first frame in which the GCaMP3 fluorescence increased and the frame where the GCaMP3.0 fluorescence returned the baseline. Ectopic calcium spikes describe those calcium spikes that did not happen within 10 seconds after pBoc.

## Supporting Information

Figure S1 Alignment of *C. elegans* PKA regulatory subunit (KIN-2a) with mouse PKA regulatory subunit (RIα). KIN-2a is well conserved. Pink color indicates identity and green color represents similarity. The positions for site A and site B, where cAMP binds, are indicated by black rectangles. The asterisk “*” indicates the Glycine residue in KIN-2a (G310), which was substituted by Aspartic acid (G310D) to make dominant negative PKA (PKA[DN]).
(TIF)

Figure S2 Alignment of *C. elegans* PKA catalytic subunit (KIN-1a) with mouse PKA catalytic subunit (Cα). KIN-1a is well conserved. Pink color indicates identity and green color represents similarity. The two asterisks “*” represent the Histidine and the Tryptophan residues in KIN-1a (H96, W205), which were substituted by Glutamine (H96Q) and Arginine (W205R), respectively, to make constitutively active PKA (PKA[CA]).
(TIF)

Figure S3 Constitutively active PKA specifically in GABAergic neurons mimics *kin-2(lf)* mutants. Quantification of the Exp step of young adult worms with the indicated genotypes. *kin-2(lf)* denotes the loss-function allele of PKA regulatory subunit, *kin-2(ce179)*. PKA[CA] denotes PKA constitutively active transgenic worms (*vjIs102* and *vjIs103*) expressing the mutated catalytic subunit *kin-1a*(H96Q, W205R) in GABAergic neurons using the *unc-47* full length promoter. The null mutants, *nlp-40(tm4085)*, were used. Means and standard errors are shown. Asterisks indicate significant differences from *nlp-40* mutants: * P<0.05, ** P<0.01 in Student's t-test.
(TIF)

Figure S4 EGL-19 and UNC-2 do not completely block ectopic calcium spikes in DVB neurons induced by constitutively active PKA. (A) Average frequency of ectopic calcium spikes in DVB neurons during a 250-second imaging period in *egl-19; unc-2* (0.0±0.00 events per 250 seconds, n = 11 animals), *PKA[CA]; egl-19; unc-2* (1.3±0.55 events per 250 seconds, n = 12 animals) and *PKA[CA]* (2.5±0.57 events per 250 seconds, n = 12 animals). (B) and (C) Quantification of the duration and amplitude of ectopic DVB calcium spikes in worms with indicated genotypes. *vjIs64*, a transgenic strain with GCaMP3 expressed in DVB neuron was used for calcium imaging. PKA[CA] represents transgenic worms with constitutively active PKA specifically expressed in GABAergic neurons (*vjIs102*). Means and standard errors are shown. Asterisks indicate significant difference from *egl-19; unc-2* mutants in (A) and between indicated groups in (C): *, P<0.05; ***, p<0.005 in Student's t-test. “n.s.” indicates no significant difference between indicated groups.
(TIF)

Video S1 Real time calcium imaging in the DVB neuron of wild type. The strain *unc-13(s69); vjIs58* was used as wild type, since *unc-13(s69)* mutants are almost paralyzed but still have normal Exp steps. *vjIs58* is an integrated transgene in which GCaMP3 is expressed in DVB neurons. One representative defecation cycle is shown. A calcium spike in the synaptic region of the DVB neuron was observed immediately before the Exp step.
(MOV)

Video S2 Real time calcium imaging in the DVB neuron of *aex-2* mutants. *unc-13(s69); vjIs58; aex-2(sa3)* was used. One representative defecation cycle is shown. No calcium spike in the synaptic region of the DVB neuron and no Exp were observed following the pBoc.
(MOV)

Video S3 Real time calcium imaging in the DVB neuron of *unc-25* mutants. The strain *unc-13(s69); unc-25(e156); vjIs58* was used. One representative defecation cycle is shown. A calcium spike in the synaptic region of the DVB neuron, but no Exp was observed following the pBoc.
(MOV)

Video S4 Real time calcium imaging in the DVB neuron of PKA[DN] transgenic animals. The strain *unc-13(s69); vjIs58; vjIs76* was used. *vjIs76* is an integrated transgene with PKA[DN] specifically expressed in GABAergic neurons. One representative defecation cycle is shown. No calcium spike in the synaptic region of the DVB neuron and no Exp were observed following the pBoc.
(MOV)

Video S5 Regular calcium spike in the DVB neuron of PKA[CA] transgenic animals. The strain *unc-13(s69); vjIs58; vjIs102* was used. *vjIs102* is an integrated transgene with PKA[CA] specifically expressed in GABAergic neurons. One representative defecation cycle is shown. A calcium spike in the synaptic region of the DVB neuron was observed immediately before the Exp step. The duration of the calcium influx was increased.
(MOV)

Video S6 Ectopic calcium spike in DVB neuron of PKA[CA] transgenic animals. The strain *unc-13(s69); vjIs58; vjIs102* was used. *vjIs102* is an integrated transgene with PKA[CA] specifically expressed in GABAergic neurons. One representative ectopic calcium spike is shown. An ectopic calcium spike in the synaptic region of the DVB neuron was observed in between cycles. Generally, the ectopic calcium spikes did not produce ectopic Exp. The duration of the calcium influx was increased.
(MOV)

Video S7 Real time calcium imaging in the DVB neuron of *unc-2* mutants in normal cycles. The strain *unc-13(s69); vjIs58; unc-2(lj1)* was used. One representative normal defecation cycle is shown. A calcium spike in the synaptic region of the DVB neuron was observed immediately before the Exp step.
(MOV)

Video S8 Real time calcium imaging in the DVB neuron of *unc-2* mutants in incomplete cycles. The strain *unc-13(s69); vjIs58; unc-2(lj1)* was used. One representative incomplete defecation cycle is shown. A weak calcium spike in the synaptic region of the DVB neuron, but no Exp was observed.
(MOV)

Video S9 Real time calcium imaging in the DVB neuron of *egl-19; unc-2* mutants in incomplete cycles. The strain *vjIs64; egl-19(n582); unc-2(lj1)* was used. *vjIs64* is an integrated transgene with GCaMP3 expressed in DVB neurons. One representative incomplete defecation cycle is shown. A weak calcium spike in the synaptic region of the DVB neuron but no Exp was observed following pBoc.
(MOV)

Video S10 Real time calcium imaging in the DVB neuron of *egl-19; unc-2* mutants in incomplete cycles. The strain *vjIs64; egl-19(n582); unc-2(lj1)* was used. One representative incomplete defecation cycle without calcium spike is shown. No calcium spike in the synaptic region of the DVB neuron and no Exp were observed following pBoc.
(MOV)

Video S11 Regular calcium spike in the DVB neuron of *PKA[CA];egl-19;unc-2* animals. The strain *vjIs64; egl-19(n582); vjIs102; unc-2(lj1)* was used. *vjIs102* is an integrated transgene with PKA[CA] specifically expressed in GABAergic neurons. One representative incomplete defecation cycle was shown. A calcium spike in the synaptic region of the DVB neuron but no Exp was observed following pBoc. The duration of the calcium spike increased.
(MOV)

Video S12 Ectopic calcium spike in the DVB neuron of *PKA[CA];egl-19;unc-2* animals. The strain *vjIs64; egl-19(n582); vjIs102; unc-2(lj1)* was used. *vjIs102* is an integrated transgene with PKA[CA] specifically expressed in GABAergic neurons. One representative ectopic calcium spike was shown. An ectopic calcium spike in the synaptic region of the DVB neuron was observed in between cycles. Generally, the ectopic calcium spikes did not produce ectopic Exp. The duration of the calcium spike increased.
(MOV)

## References

[1] Song HJ, Ming GL, Poo MM (1997) cAMP-induced switching in turning direction of nerve growth cones. Nature 388: 275–279. 9230436 10.1038/40864

[2] Ghosh-Roy A, Wu Z, Goncharov A, Jin Y, Chisholm AD (2010) Calcium and cyclic AMP promote axonal regeneration in Caenorhabditis elegans and require DLK-1 kinase. J Neurosci 30: 3175–3183. 20203177 10.1523/JNEUROSCI.5464-09.2010 PMC2921707

[3] Pifferi S, Boccaccio A, Menini A (2006) Cyclic nucleotide-gated ion channels in sensory transduction. FEBS Lett 580: 2853–2859. 16631748 10.1016/j.febslet.2006.03.086

[4] Silva AJ, Kogan JH, Frankland PW, Kida S (1998) CREB and memory. Annu Rev Neurosci 21: 127–148. 9530494 10.1146/annurev.neuro.21.1.127

[5] Mahoney TR, Luo S, Round EK, Brauner M, Gottschalk A, et al (2008) Intestinal signaling to GABAergic neurons regulates a rhythmic behavior in Caenorhabditis elegans. Proc Natl Acad Sci U S A 105: 16350–16355. 18852466 10.1073/pnas.0803617105 PMC2570992

[6] Shafer OT, Kim DJ, Dunbar-Yaffe R, Nikolaev VO, Lohse MJ, et al (2008) Widespread receptivity to neuropeptide PDF throughout the neuronal circadian clock network of Drosophila revealed by real-time cyclic AMP imaging. Neuron 58: 223–237. 18439407 10.1016/j.neuron.2008.02.018 PMC2586874

[7] Levine JD, Casey CI, Kalderon DD, Jackson FR (1994) Altered circadian pacemaker functions and cyclic AMP rhythms in the Drosophila learning mutant dunce. Neuron 13: 967–974. 7946340 10.1016/0896-6273(94)90262-3

[8] Hell JW (2010) Beta-adrenergic regulation of the L-type Ca2+ channel Ca(V)1.2 by PKA rekindles excitement. Sci Signal 3: pe33. 20876870 10.1126/scisignal.3141pe33

[9] Sassone-Corsi P (2012) The cyclic AMP pathway. Cold Spring Harb Perspect Biol 4: a011148. 23209152 10.1101/cshperspect.a011148 PMC3504441

[10] Kaupp UB, Seifert R (2002) Cyclic nucleotide-gated ion channels. Physiol Rev 82: 769–824. 12087135 10.1152/physrev.00008.2002

[11] Gloerich M, Bos JL (2010) Epac: defining a new mechanism for cAMP action. Annu Rev Pharmacol Toxicol 50: 355–375. 20055708 10.1146/annurev.pharmtox.010909.105714

[12] Skalhegg BS, Tasken K (2000) Specificity in the cAMP/PKA signaling pathway. Differential expression,regulation, and subcellular localization of subunits of PKA. Front Biosci 5: D678–693. 10922298 10.2741/skalhegg

[13] Seino S, Shibasaki T (2005) PKA-dependent and PKA-independent pathways for cAMP-regulated exocytosis. Physiol Rev 85: 1303–1342. 16183914 10.1152/physrev.00001.2005

[14] Majercak J, Kalderon D, Edery I (1997) Drosophila melanogaster deficient in protein kinase A manifests behavior-specific arrhythmia but normal clock function. Mol Cell Biol 17: 5915–5922. 9315649 10.1128/mcb.17.10.5915 PMC232439

[15] Joiner WJ, Crocker A, White BH, Sehgal A (2006) Sleep in Drosophila is regulated by adult mushroom bodies. Nature 441: 757–760. 16760980 10.1038/nature04811

[16] Reiken S, Lacampagne A, Zhou H, Kherani A, Lehnart SE, et al (2003) PKA phosphorylation activates the calcium release channel (ryanodine receptor) in skeletal muscle: defective regulation in heart failure. J Cell Biol 160: 919–928. 12629052 10.1083/jcb.200211012 PMC2173774

[17] Baba T, Sakisaka T, Mochida S, Takai Y (2005) PKA-catalyzed phosphorylation of tomosyn and its implication in Ca2+-dependent exocytosis of neurotransmitter. J Cell Biol 170: 1113–1125. 16186257 10.1083/jcb.200504055 PMC2171531

[18] Hell JW, Yokoyama CT, Breeze LJ, Chavkin C, Catterall WA (1995) Phosphorylation of presynaptic and postsynaptic calcium channels by cAMP-dependent protein kinase in hippocampal neurons. EMBO J 14: 3036–3044. 7621818 10.1002/j.1460-2075.1995.tb07306.x PMC394364

[19] Thomas JH (1990) Genetic analysis of defecation in Caenorhabditis elegans. Genetics 124: 855–872. 2323555 10.1093/genetics/124.4.855 PMC1203977

[20] Beg AA, Jorgensen EM (2003) EXP-1 is an excitatory GABA-gated cation channel. Nat Neurosci 6: 1145–1152. 14555952 10.1038/nn1136

[21] Branicky R, Hekimi S (2006) What keeps C. elegans regular: the genetics of defecation. Trends Genet 22: 571–579. 16911844 10.1016/j.tig.2006.08.006

[22] Dal Santo P, Logan MA, Chisholm AD, Jorgensen EM (1999) The inositol trisphosphate receptor regulates a 50-second behavioral rhythm in C. elegans. Cell 98: 757–767. 10499793 10.1016/s0092-8674(00)81510-x

[23] Wang H, Girskis K, Janssen T, Chan JP, Dasgupta K, et al (2013) Neuropeptide secreted from a pacemaker activates neurons to control a rhythmic behavior. Curr Biol 23: 746–754. 23583549 10.1016/j.cub.2013.03.049 PMC3651789

[24] Bargmann CI (1998) Neurobiology of the Caenorhabditis elegans genome. Science 282: 2028–2033. 9851919 10.1126/science.282.5396.2028

[25] Kim S, Govindan JA, Tu ZJ, Greenstein D (2012) SACY-1 DEAD-Box helicase links the somatic control of oocyte meiotic maturation to the sperm-to-oocyte switch and gamete maintenance in Caenorhabditis elegans. Genetics 192: 905–928. 22887816 10.1534/genetics.112.143271 PMC3522166

[26] Correll LA, Woodford TA, Corbin JD, Mellon PL, McKnight GS (1989) Functional characterization of cAMP-binding mutations in type I protein kinase. J Biol Chem 264: 16672–16678. 2550452

[27] Willis BS, Niswender CM, Su T, Amieux PS, McKnight GS (2011) Cell-type specific expression of a dominant negative PKA mutation in mice. PLoS One 6: e18772. 21533282 10.1371/journal.pone.0018772 PMC3075275

[28] Kim C, Xuong NH, Taylor SS (2005) Crystal structure of a complex between the catalytic and regulatory (RIalpha) subunits of PKA. Science 307: 690–696. 15692043 10.1126/science.1104607

[29] Li W, Ohlmeyer JT, Lane ME, Kalderon D (1995) Function of protein kinase A in hedgehog signal transduction and Drosophila imaginal disc development. Cell 80: 553–562. 7867063 10.1016/0092-8674(95)90509-x

[30] Orellana SA, McKnight GS (1992) Mutations in the catalytic subunit of cAMP-dependent protein kinase result in unregulated biological activity. Proc Natl Acad Sci U S A 89: 4726–4730. 1584809 10.1073/pnas.89.10.4726 PMC49156

[31] Schade MA, Reynolds NK, Dollins CM, Miller KG (2005) Mutations that rescue the paralysis of Caenorhabditis elegans ric-8 (synembryn) mutants activate the G alpha(s) pathway and define a third major branch of the synaptic signaling network. Genetics 169: 631–649. 15489510 10.1534/genetics.104.032334 PMC1449092

[32] Tian L, Hires SA, Mao T, Huber D, Chiappe ME, et al (2009) Imaging neural activity in worms, flies and mice with improved GCaMP calcium indicators. Nat Methods 6: 875–881. 19898485 10.1038/nmeth.1398 PMC2858873

[33] Jin Y, Jorgensen E, Hartwieg E, Horvitz HR (1999) The Caenorhabditis elegans gene unc-25 encodes glutamic acid decarboxylase and is required for synaptic transmission but not synaptic development. J Neurosci 19: 539–548. 9880574 10.1523/JNEUROSCI.19-02-00539.1999 PMC6782196

[34] Catterall WA (2011) Voltage-gated calcium channels. Cold Spring Harb Perspect Biol 3: a003947. 21746798 10.1101/cshperspect.a003947 PMC3140680

[35] Saheki Y, Bargmann CI (2009) Presynaptic CaV2 calcium channel traffic requires CALF-1 and the alpha(2)delta subunit UNC-36. Nat Neurosci 12: 1257–1265. 19718034 10.1038/nn.2383 PMC2805665

[36] Richmond JE, Weimer RM, Jorgensen EM (2001) An open form of syntaxin bypasses the requirement for UNC-13 in vesicle priming. Nature 412: 338–341. 11460165 10.1038/35085583 PMC2585764

[37] Mathews EA, Garcia E, Santi CM, Mullen GP, Thacker C, et al (2003) Critical residues of the Caenorhabditis elegans unc-2 voltage-gated calcium channel that affect behavioral and physiological properties. J Neurosci 23: 6537–6545. 12878695 10.1523/JNEUROSCI.23-16-06537.2003 PMC6740628

[38] Lee RY, Lobel L, Hengartner M, Horvitz HR, Avery L (1997) Mutations in the alpha1 subunit of an L-type voltage-activated Ca2+ channel cause myotonia in Caenorhabditis elegans. EMBO J 16: 6066–6076. 9321386 10.1093/emboj/16.20.6066 PMC1326290

[39] Steger KA, Shtonda BB, Thacker C, Snutch TP, Avery L (2005) The C. elegans T-type calcium channel CCA-1 boosts neuromuscular transmission. J Exp Biol 208: 2191–2203. 15914662 10.1242/jeb.01616 PMC1382270

[40] Arellano-Carbajal F, Briseno-Roa L, Couto A, Cheung BH, Labouesse M, et al (2011) Macoilin, a conserved nervous system-specific ER membrane protein that regulates neuronal excitability. PLoS Genet 7: e1001341. 21437263 10.1371/journal.pgen.1001341 PMC3060067

[41] Emrick MA, Sadilek M, Konoki K, Catterall WA (2010) Beta-adrenergic-regulated phosphorylation of the skeletal muscle Ca(V)1.1 channel in the fight-or-flight response. Proc Natl Acad Sci U S A 107: 18712–18717. 20937870 10.1073/pnas.1012384107 PMC2972969

[42] Light PE, Manning Fox JE, Riedel MJ, Wheeler MB (2002) Glucagon-like peptide-1 inhibits pancreatic ATP-sensitive potassium channels via a protein kinase A- and ADP-dependent mechanism. Mol Endocrinol 16: 2135–2144. 12198249 10.1210/me.2002-0084

[43] Johnstone DB, Wei A, Butler A, Salkoff L, Thomas JH (1997) Behavioral defects in C. elegans egl-36 mutants result from potassium channels shifted in voltage-dependence of activation. Neuron 19: 151–164. 9247271 10.1016/s0896-6273(00)80355-4

[44] Kaczmarek LK, Jennings KR, Strumwasser F, Nairn AC, Walter U, et al (1980) Microinjection of catalytic subunit of cyclic AMP-dependent protein kinase enhances calcium action potentials of bag cell neurons in cell culture. Proc Natl Acad Sci U S A 77: 7487–7491. 6261262 10.1073/pnas.77.12.7487 PMC350530

[45] Castellucci VF, Kandel ER, Schwartz JH, Wilson FD, Nairn AC, et al (1980) Intracellular injection of t he catalytic subunit of cyclic AMP-dependent protein kinase simulates facilitation of transmitter release underlying behavioral sensitization in Aplysia. Proc Natl Acad Sci U S A 77: 7492–7496. 6111794 10.1073/pnas.77.12.7492 PMC350531

[46] Osterrieder W, Brum G, Hescheler J, Trautwein W, Flockerzi V, et al (1982) Injection of subunits of cyclic AMP-dependent protein kinase into cardiac myocytes modulates Ca2+ current. Nature 298: 576–578. 6285199 10.1038/298576a0

[47] Wong W, Scott JD (2004) AKAP signalling complexes: focal points in space and time. Nat Rev Mol Cell Biol 5: 959–970. 15573134 10.1038/nrm1527

[48] Gancedo JM (2013) Biological roles of cAMP: variations on a theme in the different kingdoms of life. Biol Rev Camb Philos Soc 88: 645–68. 23356492 10.1111/brv.12020

[49] Brandon EP, Idzerda RL, McKnight GS (1997) PKA isoforms, neural pathways, and behaviour: making the connection. Curr Opin Neurobiol 7: 397–403. 9232801 10.1016/s0959-4388(97)80069-4

[50] Niswender CM, Willis BS, Wallen A, Sweet IR, Jetton TL, et al (2005) Cre recombinase-dependent expression of a constitutively active mutant allele of the catalytic subunit of protein kinase A. Genesis 43: 109–119. 16155866 10.1002/gene.20159

[51] Murray P, Clegg RA, Rees HH, Fisher MJ (2008) siRNA-mediated knockdown of a splice variant of the PK-A catalytic subunit gene causes adult-onset paralysis in C. elegans. Gene 408: 157–163. 18077108 10.1016/j.gene.2007.10.034

[52] Murray AJ (2008) Pharmacological PKA inhibition: all may not be what it seems. Sci Signal 1: re4. 18523239 10.1126/scisignal.122re4

[53] May RC, Plasterk RH (2005) RNA interference spreading in C. elegans. Methods Enzymol 392: 308–315. 15644189 10.1016/S0076-6879(04)92018-6

[54] Liu DW, Thomas JH (1994) Regulation of a periodic motor program in C. elegans. J Neurosci 14: 1953–1962. 8158250 10.1523/JNEUROSCI.14-04-01953.1994 PMC6577142

[55] Eastman C, Horvitz HR, Jin Y (1999) Coordinated transcriptional regulation of the unc-25 glutamic acid decarboxylase and the unc-47 GABA vesicular transporter by the Caenorhabditis elegans UNC-30 homeodomain protein. J Neurosci 19: 6225–6234. 10414952 10.1523/JNEUROSCI.19-15-06225.1999 PMC6782798

[56] Mello CC, Kramer JM, Stinchcomb D, Ambros V (1991) Efficient gene transfer in C.elegans: extrachromosomal maintenance and integration of transforming sequences. EMBO J 10: 3959–3970. 1935914 10.1002/j.1460-2075.1991.tb04966.x PMC453137

