# Supplementary figures and images for: PKA Controls Calcium Influx into Motor Neurons during a Rhythmic Behavior
Source: PLoS Genet. 2013 Sep 26;9(9):e1003831. doi: 10.1371/journal.pgen.1003831 (PMC3784516; doi:10.1371/journal.pgen.1003831)

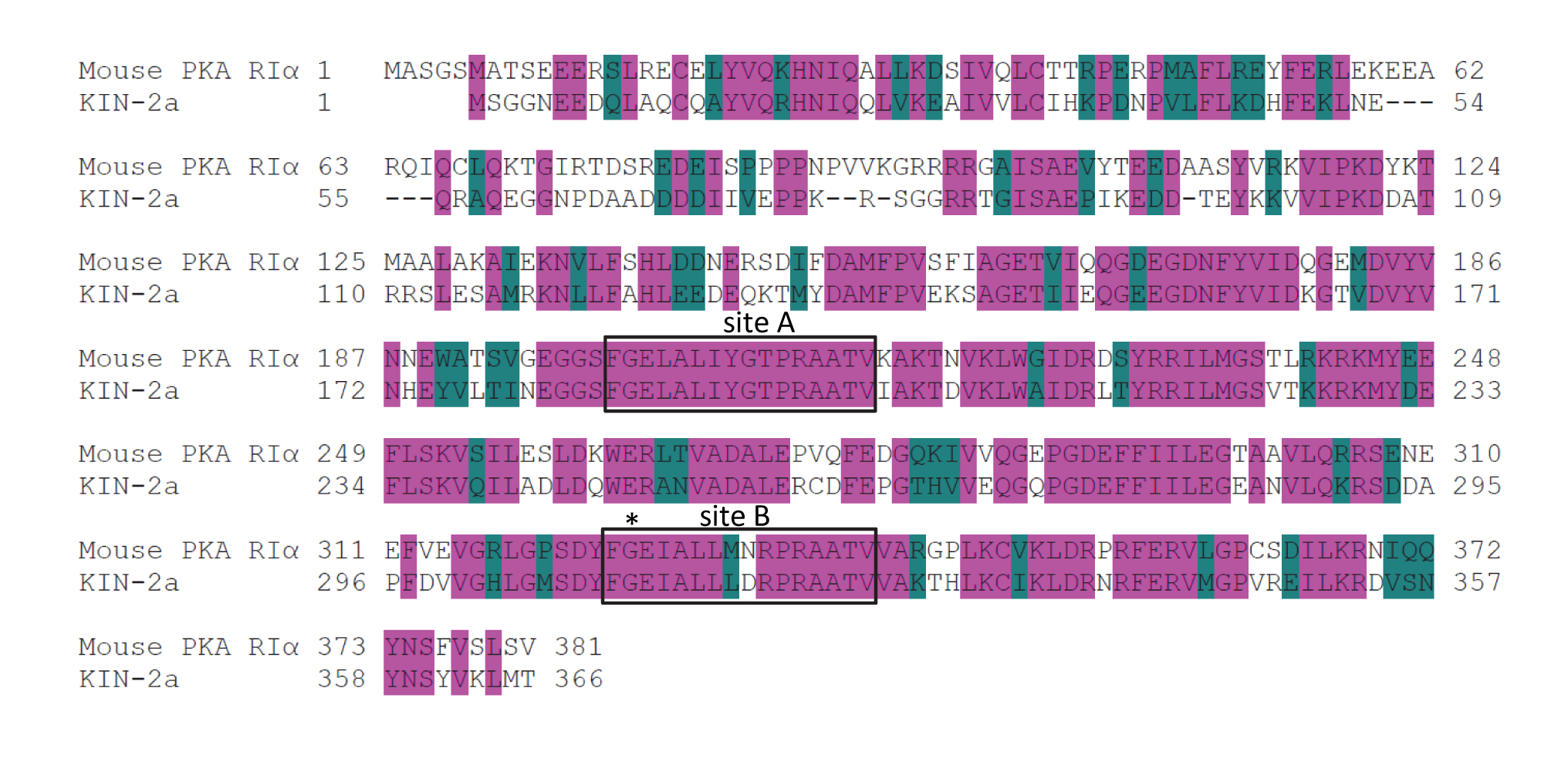

Supplement: Figure S1 — Alignment of C. elegans PKA regulatory subunit (KIN-2a) with mouse PKA regulatory subunit (RIα). KIN-2a is well conserved. Pink color indicates identity and green color represents similarity. The positions for site A and site B, where cAMP binds, are indicated by black rectangles. The asterisk “*” indicates the Glycine residue in KIN-2a (G310), which was substituted by Aspartic acid (G310D) to make dominant negative PKA (PKA[DN]). (TIF) [file pgen.1003831.s001.tif]

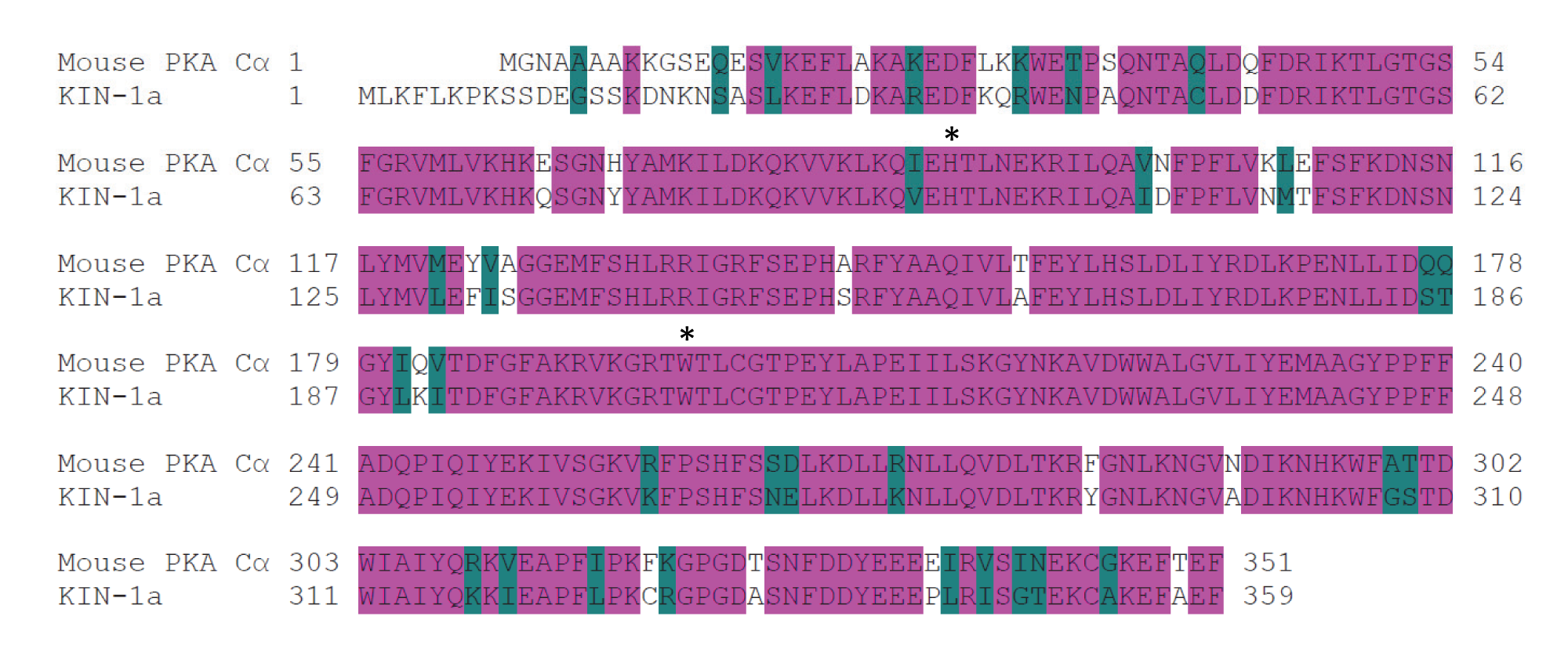

Supplement: Figure S2 — Alignment of C. elegans PKA catalytic subunit (KIN-1a) with mouse PKA catalytic subunit (Cα). KIN-1a is well conserved. Pink color indicates identity and green color represents similarity. The two asterisks “*” represent the Histidine and the Tryptophan residues in KIN-1a (H96, W205), which were substituted by Glutamine (H96Q) and Arginine (W205R), respectively, to make constitutively active PKA (PKA[CA]). (TIF) [file pgen.1003831.s002.tif]

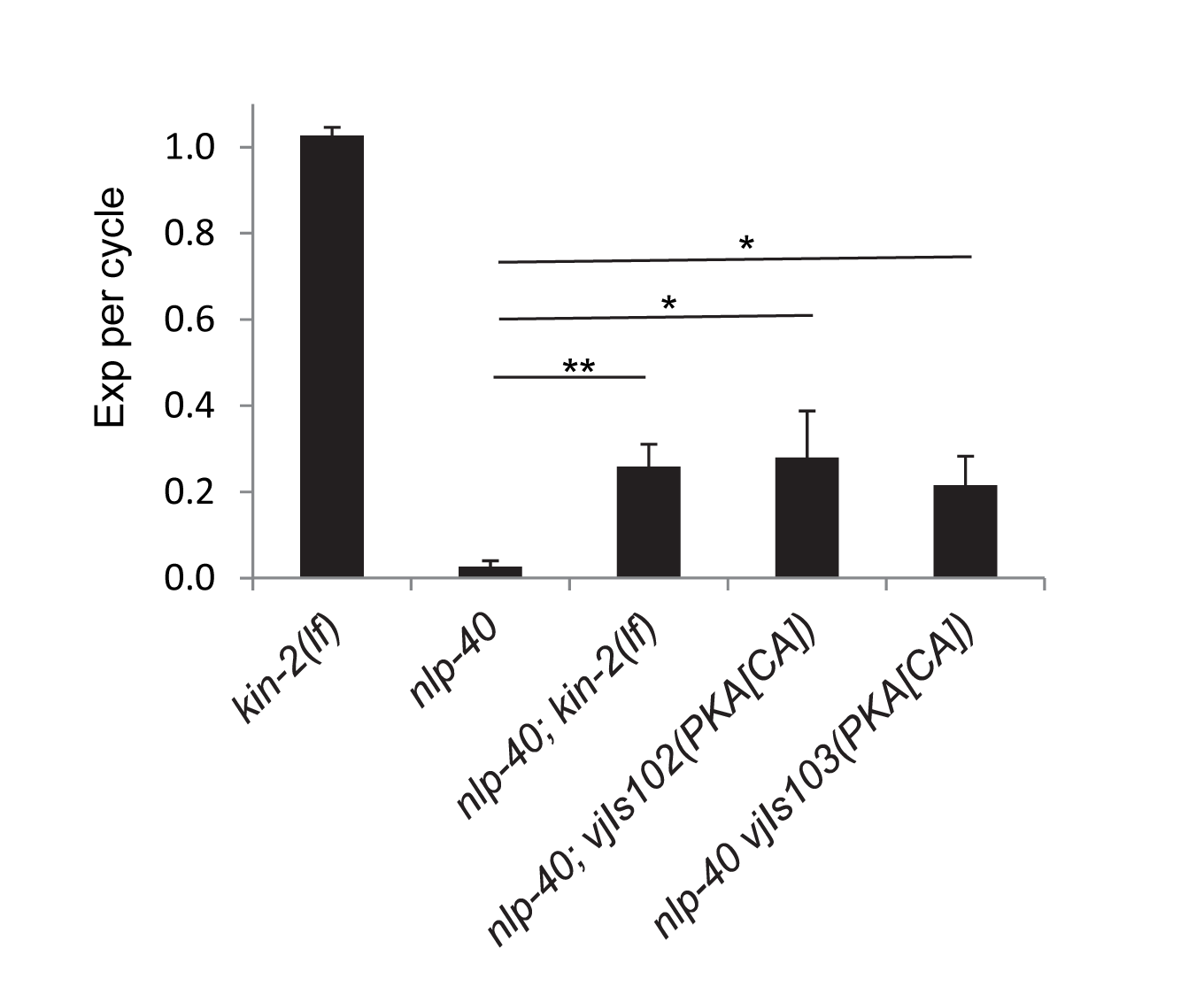

Supplement: Figure S3 — Constitutively active PKA specifically in GABAergic neurons mimics kin-2(lf) mutants. Quantification of the Exp step of young adult worms with the indicated genotypes. kin-2(lf) denotes the loss-function allele of PKA regulatory subunit, kin-2(ce179). PKA[CA] denotes PKA constitutively active transgenic worms (vjIs102 and vjIs103) expressing the mutated catalytic subunit kin-1a(H96Q, W205R) in GABAergic neurons using the unc-47 full length promoter. The null mutants, nlp-40(tm4085), were used. Means and standard errors are shown. Asterisks indicate significant differences from nlp-40 mutants: * P<0.05, ** P<0.01 in Student's t-test. (TIF) [file pgen.1003831.s003.tif]

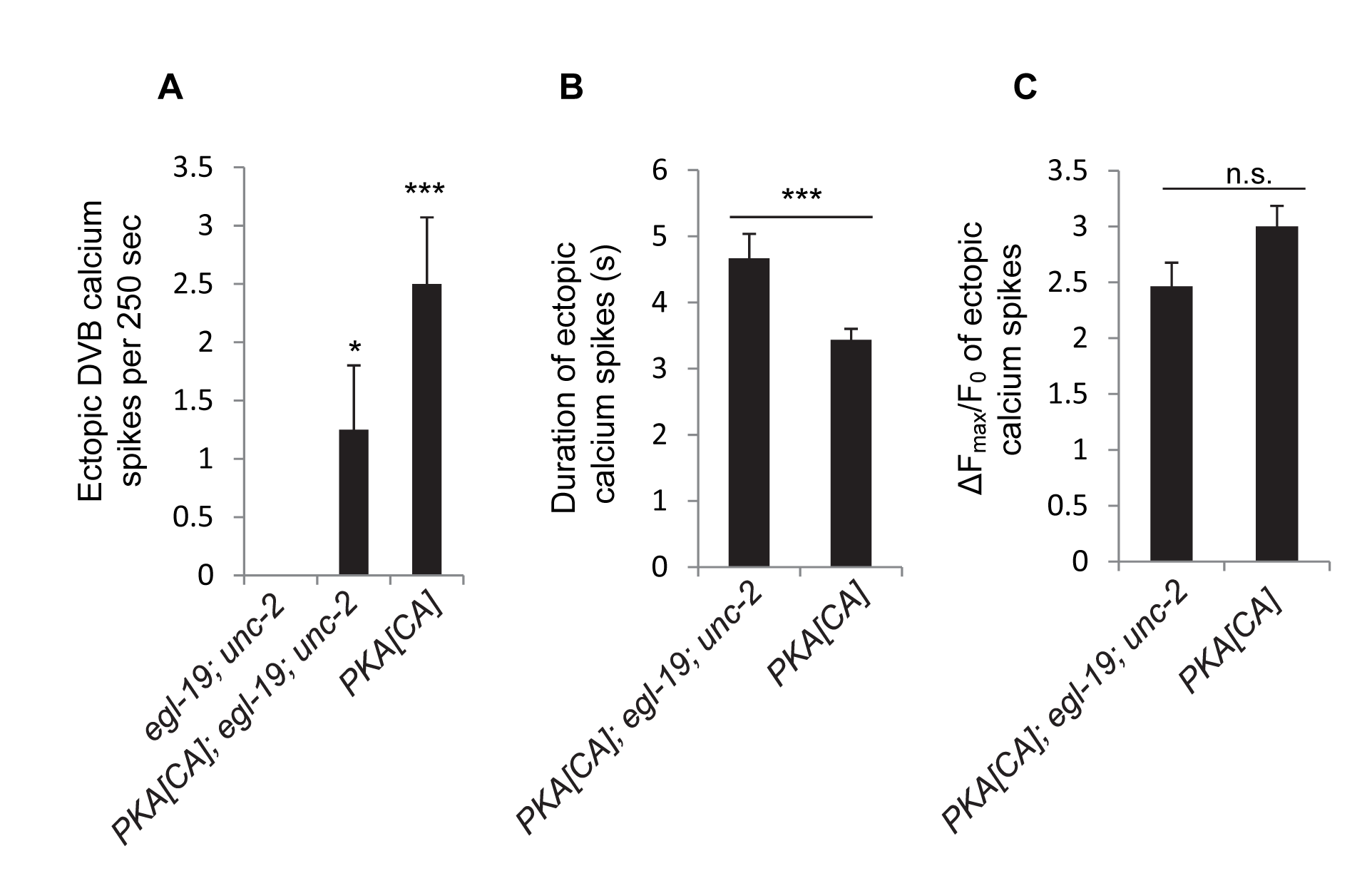

Supplement: Figure S4 — EGL-19 and UNC-2 do not completely block ectopic calcium spikes in DVB neurons induced by constitutively active PKA. (A) Average frequency of ectopic calcium spikes in DVB neurons during a 250-second imaging period in egl-19; unc-2 (0.0±0.00 events per 250 seconds, n = 11 animals), PKA[CA]; egl-19; unc-2 (1.3±0.55 events per 250 seconds, n = 12 animals) and PKA[CA] (2.5±0.57 events per 250 seconds, n = 12 animals). (B) and (C) Quantification of the duration and amplitude of ectopic DVB calcium spikes in worms with indicated genotypes. vjIs64, a transgenic strain with GCaMP3 expressed in DVB neuron was used for calcium imaging. PKA[CA] represents transgenic worms with constitutively active PKA specifically expressed in GABAergic neurons (vjIs102). Means and standard errors are shown. Asterisks indicate significant difference from egl-19; unc-2 mutants in (A) and between indicated groups in (C): *, P<0.05; ***, p<0.005 in Student's t-test. “n.s.” indicates no significant difference between indicated groups. (TIF) [file pgen.1003831.s004.tif]
